# O-GlcNAcylation of boundary element associated factor (BEAF 32) in *Drosophila melanogaster* correlates with active histone marks at the promoters of its target genes

**DOI:** 10.1080/19491034.2017.1367887

**Published:** 2017-09-14

**Authors:** Debaditya De, Satish Kallappagoudar, Jae-Min Lim, Rashmi U. Pathak, Rakesh K. Mishra

**Affiliations:** a CSIR-Centre for Cellular and Molecular Biology, Hyderabad, India; b Department of Chemistry, Changwon National University, Changwon, Gyeongnam, South Korea

**Keywords:** post-translational modification, phosphorylation, O-GlcNAcylation, TSS, H3K4me3

## Abstract

Boundary Element-Associated Factor 32 (BEAF 32) is a sequence specific DNA binding protein involved in functioning of chromatin domain boundaries in *Drosophila*. Several studies also show it to be involved in transcriptional regulation of a large number of genes, many of which are annotated to have cell cycle, development and differentiation related function. Since post-translational modifications (PTMs) of proteins add to their functional capacity, we investigated the PTMs on BEAF 32. The protein is known to be phosphorylated and O-GlcNAcylated. We mapped O-GlcNAc site at T91 of BEAF 32 and showed that it is linked to the deposition of active histone (H3K4me3) marks at transcription start site (TSS) of associated genes. Its role as a boundary associated factor, however, does not depend on this modification. Our study shows that by virtue of O-GlcNAcylation, BEAF 32 is linked to epigenetic mechanisms that activate a subset of associated genes.

## Introduction

Chromatin insulators or boundaries are specialized chromatin elements that prevent spurious enhancer-promoter interactions and nullify the chromosomal position effect on the genes which they bracket.^1-3^ Insulators were originally identified in *Drosophila melanogaster* and *scs* and *scs'* are the first such DNA elements discovered.^2^ These elements are found to be bracketing the HSP70 genes at the 87A7 cytological locus of *Drosophila*. Depending on the insulator binding protein (IBP) that is responsible for its activity, distinct families of insulators have been defined.^4^ In *Drosophila* 5 major families have been described depending on the IBPs, namely, Boundary Element Associated Factor 32 kDa (BEAF 32), Zeste-white 5 (Zw5), Suppressor of Hairy-wing [Su(Hw)], GAGA factor (GAF) and CCCTC-binding factor (dCTCF). The function of these insulators further depends on cofactors, like centrosomal protein 190 (CP190) and Mod(mdg4), which through protein-protein interaction bridge the contact between distant genomic regions.^5,6^ The IBPs share several common molecular mechanisms of function. For example, all the IBPs alter accessibility of associated chromatin and the long-range interactions they establish with distant elements leads to compartmentalization of chromatin. The most widely accepted model for insulator function posits that distant elements cluster together resulting in looping of the intervening DNA that carry multiple genes and their respective regulatory elements inside the loop. The loops thus act as an independent domain.^7,8^

Parallel investigations on chromatin organization using 3C-based methods followed by high-throughput sequencing have revealed that chromosomes are divided into self-interacting domains known as topologically associated domains (TADs).^9^ In *Drosophila*, TAD boundaries are enriched with active chromatin marks and constitutively transcribing active genes. Interestingly, IBPs like BEAF 32, GAF and dCTCF are also enriched at TAD boundaries emphasizing a close link between transcription, insulators/IBPs, TADs and chromatin organization.^10-12^ By virtue of their long-range interactions, IBPs possibly cluster active gene-dense regions away from silenced regions and in the process, contribute to the establishment of TADs. While mechanistic details of chromatin organization are still emerging, it is clear from genome-wide distribution of IBPs that, along with the classical insulator activity, they play other important functions *in situ*.^4,13-15^ Apart from being present at borders of heterochromatin domains, the IBPs were found to bind near promoters of active genes. In *Drosophila melanogaster* BEAF 32 and dCTCF binding sites are specifically enriched close to promoter. Immunofluorescence studies done on *Drosophila* polytene chromosome showed that BEAF 32 binds to many interband and band/interband boundaries.^16,17^ Studies show that 85% of BEAF 32 binding sites are enriched within 300 base pairs upstream or downstream of transcription start sites (TSS) and more so located predominantly between closely spaced, differentially expressed, head to head gene pairs.^5,18^ Of the 2 isoforms of the protein, namely BEAF 32A and BEAF 32B, the latter has a more dominant role in binding to chromatin.^18^ During embryogenesis, depletion of BEAF 32 from its chromatin binding sites using a transgenic dominant-negative form of the protein results in lethality.^19^ Phenotypically BEAF 32 has a major role in the regulation of genes which effect cell cycle.^20^ This is supported by the observation that depletion of BEAF 32 leads to alteration of cell cycle and chromosome segregation defect.^20^ Although, maternally deposited BEAF 32 is enough for an embryo to develop into an adult, BEAF 32 knock out flies are sickly and exhibit female sterility due to defective oocyte development.^21^ These studies suggest that BEAF 32 is important for development and differentiation processes.

ChIP-seq and ChIP-chip studies and modENCODE data for various histone marks in *Drosophila melanogaster* have found a relationship between insulators sites and the histone marks that are found in the adjacent regions. Analysis of such genomic regions have shown that presence of IBPs helps to sculpt the chromatin landscape of the nearby regions. BEAF 32, in its role as a classical IBP, can restrict the spreading of H3K27me3 at a few Polycomb target regions and prevent repressive histone methylation of adjacent genes.^22^ Intriguingly, most of the BEAF 32 bound regions are also enriched in activating histone marks such as H3K4me3.^18^ BEAF 32 performs these functions by associating with various histone modifiers and chromatin re-modelers. Thus, BEAF 32 along with CP190 controls nucleosome organization around BEAF 32 element binding sites^23^ and BEAF 32 with the help of dMes-4 leads to recruitment of HATs.^24^ Even at the differentially regulated head to head genes separated by BEAF 32 binding site, hyperacetylation of histones at TSS has been reported. Most of these marks are BEAF 32 dependent and involved in an active state of expression of one gene of the gene pair. Although such BEAF 32 dependent activation of expression of genes has been studied to some extent, the properties of the protein that makes it capable of recruiting various histone marks at TSS of different genes has not been elucidated.

Several PTMs have been implicated on IBPs as modifications that can add regulatory features to proteins. For example, while phosphorylation and SUMOylation of CTCF is linked to its repressive activity, poly(ADP-ribosyl)ation is essential for it to act as an insulator protein.^25-27^ Similarly, phosphorylation of GAF reduces its DNA binding affinity.^28^ In yet another example, SUMOylation of Mod(mdg4) and CP190 inhibits the long range interaction of insulator complexes which in turn regulates the establishment of chromatin domains.^29^

PTMs of BEAF 32 remain a relatively unexplored domain. Previous studies have revealed that BEAF 32 is phosphorylated and O-GlcNAcylated.^16,17,30^ But neither of the PTMs has been mapped on the protein, nor have they been investigated in detail. In the current study, we have found a link between O-GlcNAcylation of BEAF 32 and regulation of expression of associated genes. Interestingly many of the effected genes are associated with cell cycle and differentiation related functions. We mapped the O-GlcNAcylation site of BEAF 32 by mass spectrometry and then using site-directed mutagenesis (SDM) we mutated the site so that it does not support O-GlcNAcylation anymore. Using this mutated version, we have biochemically and functionally characterized BEAF 32 that lacks O-GlcNAcylation. Our results show a direct link between O-GlcNAcylation of BEAF 32 and increased expression of a subset of associated genes that are linked to cell cycle and differentiation related functions. This is achieved by deposition of H3K4me3 at the promoters of these genes. Our experiments also show that in actively dividing cells/differentiating tissues of *Drosophila melanogaster*, BEAF 32 is O-GlcNAcylated but in non-dividing and differentiated tissue the protein is devoid of this modification. For the first time, here we have established the role of PTM of an IBP in active transcription of a certain set of genes involved in specific biologic processes. Our study throws some light on how a PTM of BEAF 32 converts a widely-expressed IBP into a highly specific transcriptional regulator.

## Results

### PTM of BEAF 32 modulate its DNA binding and nuclear matrix localization

**Figure 1.**
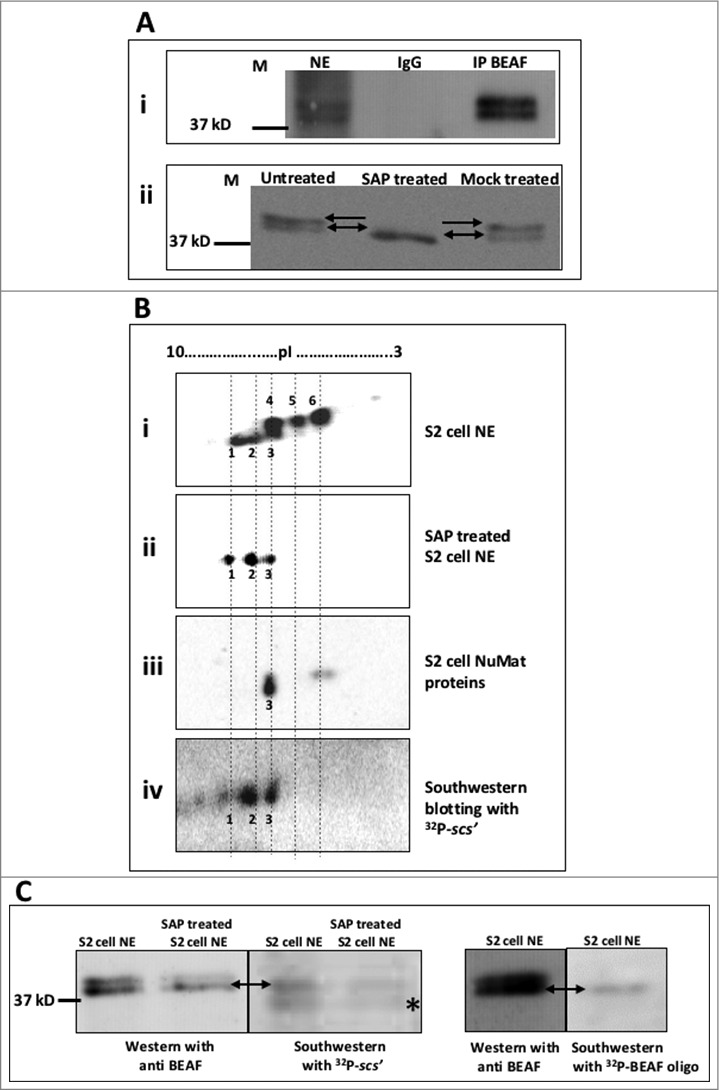
BEAF 32 is phosphorylated and a specific isoform binds to DNA and NuMat. A. i. Phosphorylated as well as dephosphorylated BEAF 32 get immuno-precipitated from nuclear extract (NE) of S2 cells. Immuno-precipitated fractions with anti BEAF (IP BEAF) or IgG were resolved using 12% SDS-PAGE and western blotted with anti BEAF. **ii**. Phosphatase treatment abolishes the upper band (phosphorylated form) of BEAF 32 doublet, marked by single head arrow. Dephosphorylated form, marked by double head arrow, is present in all and enriched in SAP treated lane at the expense of phosphorylated form. S2 cell NE (untreated), Shrimp Alkaline Phosphatase treated NE (SAP treated) and 1X SAP buffer treated NE (Mock treated) were western blotted and probed with anti BEAF. B. Dephosphorylated BEAF 32 binds to DNA and localizes to NuMat. NE or NuMat proteins from S2 cells, was electro-focused on pH 3–10 NL strip and then resolved using 12% SDS-PAGE. Western blots were probed with anti BEAF. For southwestern analysis, the same blot used for western was probed with ^32^P-labeled *scs'* DNA to reveal the DNA binding variants i. PTM variants of BEAF 32 in S2 cell extract resolved as 6 distinct spots (1–6) in 2D PAGE. **ii**. SAP treatment of the S2 extract abolished the upper spots (4–6). **iii**. One of the dephosphorylated variant (spot 3) was present in NuMat. **iv**. Dephosphorylated variants of BEAF 32 (spots 2 and 3) bound to DNA. C. Dephosphorylated BEAF 32 binds to DNA. Untreated or SAP treated S2 cell NE, were resolved on 12% SDS-PAGE. Western blot was probed with anti BEAF, followed by southwestern with ^32^P-labeled *scs'* DNA. The band marked with * represents a non-specific protein bound by *scs'* which does not show up when an oligo carrying only BEAF 32 binding site is used as a probe for southwestern.

Previous studies have shown that BEAF 32 is phosphorylated.^16^ Theoretical molecular weight of the protein is 32kD, but it runs as a doublet close to 38kD in a SDS-PAGE. The doublet has been attributed to phosphorylation. Immuno-precipitation of BEAF 32 from S2 cell nuclear extract (NE), showed that both the forms (phosphorylated as well as dephosphorylated BEAF 32) were present in the nucleus (Fig. 1A i). Using Shrimp alkaline phosphatase (SAP) we reconfirmed that the upper band in the doublet in a western blot analysis represents phosphorylated form of BEAF 32 as it disappears upon phosphatase treatment (Fig. 1A ii).

By performing 2-dimensional (2D) PAGE followed by western blotting with anti BEAF, we observed that several variants of BEAF 32 exist, that differ in their molecular weight and pI values (spots 1–6 in Fig. 1B i). Treatment of S2 cell NE with phosphatase resulted in abolition of the upper spots 4, 5 and 6. However the 3 lower spots 1, 2 and 3 with different pI values, still remain, indicating that PTM(s) other than phosphorylation exists on the protein (Fig. 1B ii). As these isoforms are not abolished by phosphatase treatment, we speculate that the PTM may be acetylation of a lysine residue that leads to masking of positive charge on lysine, imparting net negative charge to the protein. These PTM isoforms of BEAF 32 need to be investigated further.

We then explored the biologic relevance of BEAF 32 PTM variants. As BEAF 32 is known to be retained in nuclear matrix (NuMat), we asked if a particular variant was enriched in this nuclear sub-structure. NuMat proteins were isolated from S2 cells and analyzed by 2D western to find out which of the 6 variants associates with NuMat. On comparison, we observed that predominantly spot 3 as well as some amount of spot 4 and 6 are the BEAF 32 variants that associated with NuMat (Fig. 1B iii). Further, as BEAF 32 is a known DNA binding protein we wanted to know if a particular variant is responsible for its DNA binding activity. Using *scs'* DNA element which is one of BEAF 32 targets, we performed southwestern blotting with S2 cell NE resolved on 2D PAGE and ^32^P-labeled *scs'* DNA. We found that spots 2 and 3 bind to DNA (Fig. 1B iv). We further analyzed this observation with southwestern blotting's on 1D SDS-PAGE (Fig. 1C and Supplementary Figure 1). Untreated and SAP treated S2 cell NE were resolved on SDS-PAGE and probed with anti BEAF. The same blot was processed for southwestern with ^32^P-labeled *scs'* DNA. Southwestern confirmed that the lower dephosphorylated isoform of BEAF 32 binds to *scs'* DNA (Fig. 1C). However, the *scs'* DNA bound to another protein as well (marked by *), which runs below BEAF 32 in SDS-PAGE. We then used a 42bp dsDNA that carries BEAF 32 recognition motif only, as a probe (Supplementary Table 1). This sequence bound only to the dephosphorylated isoform of BEAF 32 (Fig. 1C). Interaction of *scs'* and BEAF 32 is specific as the protein does not bind to a labeled non-specific probe (BEAF 32 protein coding sequence, Supplementary Figure 1). Based on southwestern blots, we conclude that in S2 cells the dephosphorylated isoform of BEAF 32 binds to DNA. When resolved on a 2D gel, the spot numbered 3, which is a dephosphorylated variant of BEAF 32, has DNA as well as NuMat binding capability.

**Table 1.** List of the loci and associated genes tested for their expression level and H3K4me3 status in the context of wt and T91A BEAF 32B.

Sl. No.	Loci	Reference region (H3K4me3 enriched/BEAF bound)	Adjacent Gene (ID)	Biological Process
1	SG1	chr2L:1128723…1129294	Pino (CG4710)	Olfactory behavior; Response to DNA damage stimulus
2	SG2	chr2L:20796600… 20797631	Varicose (CG9326)	Tracheal system development; Septate junction assembly
3	SG3	chr2L:17472953…17474026	Sgt (CG5094)	Regulation of protein oligomerization and protein folding
4	SG4	chrX:10793510…10793850	CG1582	RNA processing
5	SG5	chrX:20059900…20060628	CG9577	Fatty acid β-oxidation; Metabolic process
6	SG6	chr3L:18665491…18667286	GNBP2 (CG4144)	Carbohydrate metabolic process; Innate immune response
7	IG1	chr3L:18793640… 18795073	CG14073	Wing disk dorsal/ventral pattern formation
8	IG2	chrX:12656911… 12659097	CG12717	Neuron axonogenesis; Dendritic spine morphogenesis; Imaginal disk derived wing morphogenesis
9	IG3	chrX:11613662… 11614618	pretaporter (CG1837)	Regulation of apoptotic cell clearance
10	IG4	chr3L:4138834…4139271	Ras opposite (CG15811)	Mitotic cytokinesis; Synaptic transmission; Neurotransmitter secretion; Dendrite development; Ovarian follicle cell development; Oskar mRNA localization
11	IG5	chr3R:2233433… 2232410	grappa (CG42803)	Regulation of cell cycle; Chromatin silencing
12	IG6	chr3R:10566399…10568000	eff (CG7425)	Mitotic cell cycle; Spermatid development; Germ-line stem cell maintenance; Neurogenesis; Compound eye morphogenesis; Chromosome organization
13	IG7	chrX:15751316…15752560	Myb (CG9045)	Mitotic cell cycle; Centriole replication; Chromosome condensation; Spindle organization; Cell proliferation; Regulation of glucose metabolic process
14	IG8	chrX:15356264…15356811	shtd (CG9198)	Mitotic cell cycle; Compound eye development
15	IG9	chrX:16530969…16532559	Rho kinase (CG9774)	Mitotic cell cycle; Morphogenesis of embryonic epithelium; Actin cytoskeleton organization; Oocyte development; Rho protein signal transduction; Axonogenesis; Myoblast fusion
16	IG10	chr2L:13828944…13830545	Orc5 (CG7833)	Mitotic chromosome condensation; Spindle organization; DNA replication

### T91 is the O-GlcNAc site in BEAF 32B

Apart from phosphorylation, BEAF 32 has also been reported to be O-GlcNAcylated. Both the bands of the protein doublet are retained on wheat germ agglutinin (WGA) indicating that phosphorylated as well as dephosphorylated BEAF 32 can bind to the lectin with high affinity.^30^ This observation suggested that BEAF 32 is O-GlcNAcylated irrespective of its phosphorylation status. However, the O-GlcNAc site in the protein has not been mapped, and its biologic relevance has also not been elucidated till now.

**Figure 2.**
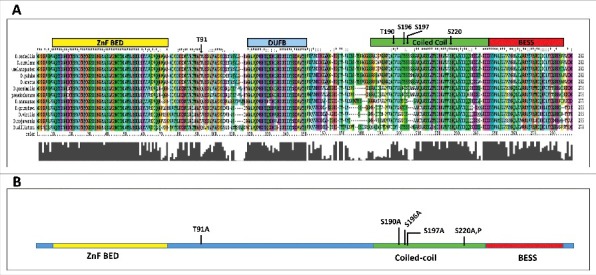
Multiple sequence alignment of BEAF 32B. A. Multiple sequence alignment was done to look for the conserved residues of BEAF 32B across all 12 *Drosophila* species. Domains have been marked using *D. melanogaster* BEAF 32B sequence as reference. Residues selected for site directed mutagenesis (SDM) are marked. B. Residues selected for SDM are shown in context of protein domains of BEAF 32B. O-GlcNAc site identification by Fourier Transform Mass Spectrometer and MS/MS without collision energy were based on fragmentation spectrum of peptides Tg^91^LREPLR and AKTg^91^LR.

With the intention to find out the residues that may have potential for O-GlcNAc attachment in BEAF 32, we used the *in silico* tool YinOYang 1.2 (http://www.cbs.dtu.dk/services/YinOYang/) to analyze the protein sequence. As most of the BEAF 32 bound sites in the *Drosophila* genome are occupied by the isoform 32B, we chose the isoform for further analysis. O-GlcNAcylation of BEAF 32 could be important in other species of *Drosophila*. To find out how conserved the predicted residues were across other *Drosophila* species, we did multiple sequence alignment of BEAF 32B in 12 sequenced *Drosophila* species and looked for conservation of the predicted serine and threonine residues (Fig. 2A). This alignment showed that most of the conserved residues that can support O-GlcNAcylation are in the coiled-coil region which is important for protein-protein interactions. Using the analysis, we selected 4 conserved residues that had high potential for O-GlcNAc modification. These serine (S) or threonine (T) residues at position T190, S196, S197 and S220 were mutated individually to alanine (A) (Fig. 2B). As alanine lacks the -OH group in the side chain, it does not support O-GlcNAc attachment. The amino acid residue S220 was mutated to both alanine (A) and proline (P) to create 2 different mutants. Mutation S220P was created as introduction of proline, a known helix breaker, in the coiled-coil region of the protein might perturb the structure (Fig. 2B). These mutant proteins were used for further analysis.

Along with theoretical prediction, we wished to map the O-GlcNAcylated site proteomically. To map the O-GlcNAcylated residues, a 6XHis tagged BEAF 32B protein was overexpressed in Sf9 cells using baculoviral expression system. Expressed protein was affinity purified using Ni-NTA column, digested with trypsin and resulting peptides were analyzed by reversed phase liquid chromatography coupled with tandem mass spectrometry (RP-LC-MS/MS) using the parent mass list method. Using the method, we identified several peptides with less than 0.60 Sf, covering 74.1% of BEAF 32B protein sequence (Supplementary Figure 2A). Two O-GlcNAc modified peptides for same site with one internal miss cleavage were identified between Ala89 and Arg93 (AKTgLR) and between Thrg91 and Arg97 (TgLREPLR) with low Sf score. To confirm the site, the target peptides were analyzed by LC-MS/MS with parent ion monitoring using Fourier Transform MS/MS without collision energy to measure mass accuracy of the parent ion from FT mass spectrum. The FT MS spectrum of the peptide between Thrg91 and Arg97 (TgLREPLR) shows the doubly charged O-GlcNAc peptide with 6.8ppm mass accuracy (Supplementary Figure 2B). Supplementary Figure 2Cshows the fragmentations of the O-GlcNAc modified peptide and neutral loss of GlcNAc from the peptide both singly and doubly charge states. Another peptide between Ala89 and Arg93 (AKTgLR), which has same O-GlcNAc site at Thrg91, was also identified by the fragmentation of the peptide and neutral loss of GlcNAc from the peptide (Supplementary Figure 2D). However, this peptide was not detected from FT MS spectrum by the parent ion monitoring of MS/MS. The mass spectrometry analysis thus identified threonine at 91 (T91) as one of the O-GlcNAc modified site with confidence. We thus included T91A mutant in our study.

**Figure 3.**
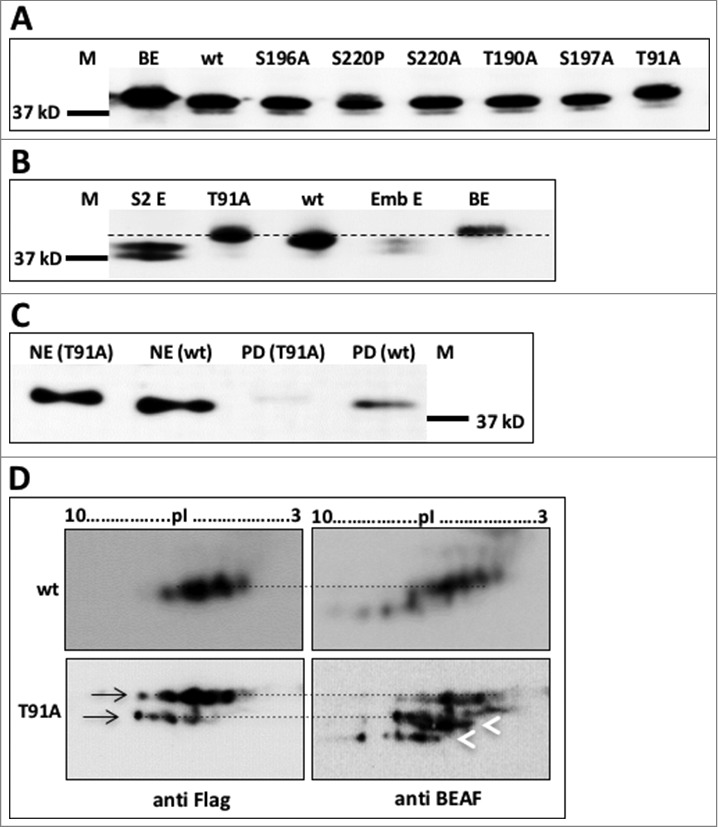
T91A mutant protein has different mobility. A. T91A mutant migrates slower than other mutants. wt and BEAF 32B mutants were expressed in S2 cells. After 24 hours of transfection, cell lysates were resolved using 8–15% gradient SDS-PAGE and analyzed by western blotting with anti-FLAG. T91A mutant migrates slower than wt, in a manner similar to bacterially expressed protein. M – Marker (37 kDa), BE – Bacterially expressed FLAG-tagged BEAF 32B, wt, S196A, S220P, S220A, T190A, S197A, T91A – lysates from S2 cells exogenously expressing wt and mutant FLAG-tagged BEAF 32B proteins. B. T91A mutant migrates slower than native BEAF 32. Western blotting was performed on proteins resolved using 8–15% gradient SDS-PAGE with anti BEAF. M – Marker (37 kDa), S2 E – S2 cell extract, T91A and wt – Extract of S2 cells expressing exogenous T91A mutant protein and wt BEAF 32B protein, Emb E – Embryonic extract, BE – Bacterially expressed BEAF 32B protein. C. T91A is the only O-GlcNAcylation site for BEAF 32B. Pull down using WGA agarose beads from NE of S2 cells transfected with either wt or T91A mutant BEAF 32B shows that T91A BEAF 32B does not get pulled down (PD) by WGA beads suggesting lack of O-GlcNAcylation. T91A PD and wt PD are the WGA pull down fraction from the respective extracts. Blot was probed with anti-FLAG. D. T91A mutant migrates slower than native BEAF 32B, but has similar phosphorylated variants. Protein extracts from S2 cells expressing FLAG-tagged wt or T91A mutant protein were resolved using 2D-gel and analyzed by sequential western blotting with anti-FLAG followed by anti BEAF. FLAG-tagged wt protein migrates at par with phosphorylated isoforms of native protein.

The wild type (wt) and the 6 mutant BEAF 32B coding sequences (5 predicted by *in silico* analysis and one mapped proteomically) were cloned into pFPc19 vector where polycomb promoter drives the expression of the mutant proteins. The expressed proteins also get tagged with FLAG-polypeptide.^31^ The plasmids capable of expressing wt and mutant BEAF 32B proteins were transfected into S2 cells individually. After 24 hrs post-transfection, proteins were extracted from the transfected cells and analyzed by western with anti BEAF. To have a fair comparison of mobility, we included bacterially expressed Flag-tagged BEAF 32B in an adjacent lane. All the extracts were loaded onto 8–15% gradient PAGE, transferred to PVDF membrane and probed with anti-FLAG (Fig. 3A). For reasons still not known, we notice that the exogenously expressed FLAG-tagged proteins migrate predominantly in a manner similar to the upper phosphorylated form of BEAF 32 doublet. We found that only T91A mutant shows a difference in mobility when compared with wt BEAF 32B. Mobility of T91A mutant protein was slower, and was equivalent to that of the bacterially expressed BEAF 32B protein (Fig. 3B). The FLAG-tagged wt BEAF 32B has slightly lower mobility than endogenous BEAF 32 from embryos and S2 cells because of the presence of Flag polypeptide which adds 1kDa to the molecular weight of the exogenously expressed wt BEAF 32B. Overall, the results indicate that mutation of T91 prevents O-GlcNAcylation and the unmodified protein behaves like bacterially expressed BEAF 32B. Interestingly, lack of modification resulted in lower mobility. Other mutants moved normally, in a manner similar to wt protein, indicating that no site other than T91 is O-GlcNAcylated in BEAF 32B.

To confirm that T91 is indeed the only site that supports O-GlcNAcylation, NE was prepared from S2 cells expressing T91A mutated and the wt BEAF 32B protein. Using wheat germ agglutinin (WGA) agarose beads, O-GlcNAcylated proteins were pulled down, separated on a 12% PAGE and transferred to PVDF membrane. Western blot with anti-FLAG showed that BEAF 32B can be detected in the pull down from NE prepared from cells expressing wt BEAF 32B protein and it is absent in the pull down done from NE prepared from cells expressing T91A mutant protein (Fig. 3C). This confirmed that T91 is the only site in BEAF 32B with O-GlcNAcylation mark. We did observe a very faint BEAF 32B band even in the T91A pull down. This might be due to the ability of T91A mutant to form complex with endogenous BEAF 32 which has O-GlcNAcylation and thus gets pulled down by WGA beads.

Further, NE from S2 cells expressing FLAG-tagged wt or T91A mutant protein were analyzed by 2D western blotting. Here the FLAG-tagged wt or T91A mutant proteins were detected with anti FLAG, and native BEAF 32 was detected by re-probing the same blot with anti BEAF (Fig. 3D). We observed that the FLAG-tagged wt protein migrates at par with the phosphorylated isoforms of native protein variants. On the other hand, the variants corresponding to T91A mutant protein (marked by black arrows) ran slower than the native protein variants (marked by white arrowheads). However, the exogenously expressed FLAG-tagged wt or T91A mutant protein shows increased number of spots that differ in pI value. This indicates that the exogenously expressed proteins may be phosphorylated differently as compared with native protein and exogenous expression somehow results in increased phosphorylation of the expressed proteins. However, the results suggest that O-GlcNAcylation of BEAF 32B is independent of phosphorylation, as removal O-GlcNAc does not affect phosphorylation efficiency of the protein.

### Functional characterization of O-GlcNAcylation of BEAF 32B

#### (i) O-GlcNAc has no role in localization of BEAF 32B *in vivo*

**Figure 4.**
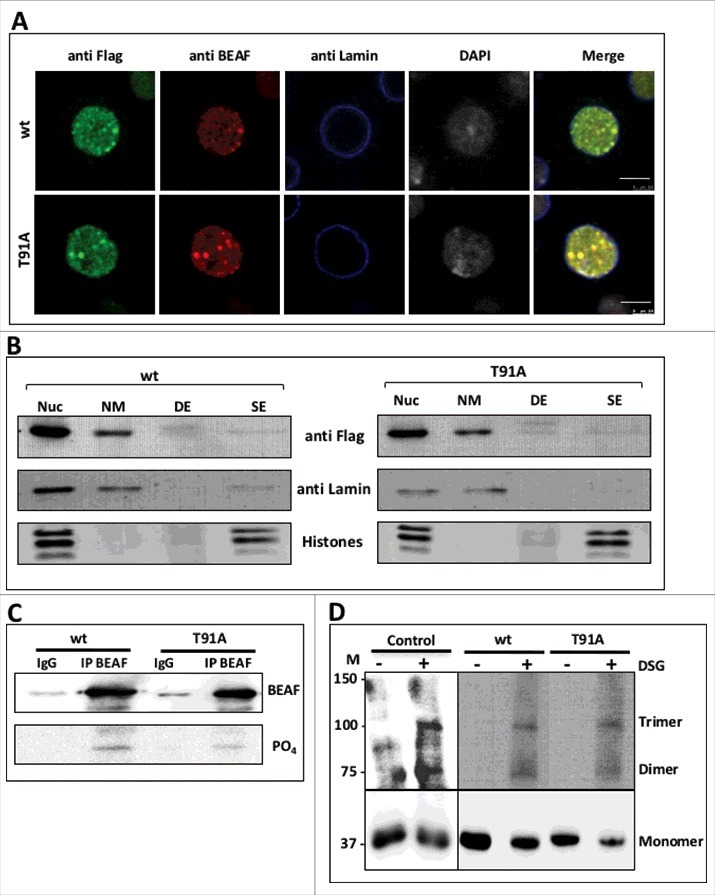
Characterization of T91A mutant protein. A. T91A mutation does not affect localization of BEAF 32. FLAG-tagged wt and T91A BEAF 32B protein were expressed in S2 cells. The exogenously expressed proteins were stained using anti FLAG and overlaid with native BEAF 32 visualized by anti BEAF. Nuclear lamina was stained with anti Lamin and DNA was stained with DAPI. The T91A mutant protein as well as the exogenously expressed wt protein colocalize with native BEAF 32. Scale Bar – 5 µM. B. T91A mutant protein associates with NuMat. NuMat protein fractions were prepared from S2 cells expressing FLAG tagged wt and T91A BEAF 32B proteins. Western blotting with anti-FLAG was performed with total nuclear proteins (Nuc), NuMat proteins (NM), DNaseI extracted proteins (DE) and salt extracted proteins (SE). The same blots were re-probed with anti Lamin and anti His4 to show quality of NuMat preparation. C. T91A mutant BEAF 32B is efficiently phosphorylated *in vitro.* Bacterial extract containing wt and T91A BEAF 32B proteins were kinased *in vitro* in the presence of gamma labeled ^32^P-ATP and S2 cell extract. Proteins were pulled down with anti BEAF and western blotted with anti BEAF (BEAF panel). Following the western, same blot was exposed for phosphor imaging to obtain the image of phosphorylated proteins, D. T91A mutant protein multimerizes in a manner similar to wt protein. Extract from control S2 cells and S2 cell expressing FLAG-tagged wt and T91A BEAF 32B were cross-linked with DSG for 20 minutes. The control and cross-linked proteins were resolved by 6% SDS-PAGE, and western blotted with anti BEAF for control S2 cell extract and anti-FLAG for transfected S2 cell extracts. Lower half of the blot was cut and developed for lesser time than the upper half to balance the signal intensity.

FLAG-tagged wt, T91A mutant as well as other mutant BEAF 32B proteins were expressed in S2 cells. The cells were immuno-stained with anti-FLAG to visualize the mutant protein. Native BEAF 32 was visualized with anti BEAF. Our results with western blotting in previous section has shown that exogenously expressed FLAG-tagged wt and mutant BEAF 32B proteins are predominantly phosphorylated as they are detected as a single band running in a manner similar to the upper phosphorylated isoform of the native BEAF 32 doublet. Further, the southwestern assay suggests that lower dephosphorylated isoform of the native protein doublet binds to DNA. Taken together these results raise a concern that the exogenously expressed proteins may not bind to target DNA sequence. However, this is not the case and co immuno-staining with anti FLAG and anti BEAF show that FLAG-tagged exogenously expressed wt or mutant BEAF 32B proteins co-localize very well with native BEAF 32. FLAG-tagged wt and mutant BEAF 32B proteins, both co-localize with native BEAF 32 with Pearson co-relation coefficient value of 0.91 and 0.90 respectively (Fig. 4A and Supplementary Figure 3A). This suggested that the FLAG-tagged wt and T91A BEAF 32B proteins multimerize with native BEAF 32 isoforms and get recruited to target native genomic loci. Lack of O-GlcNAc does not disrupt its localization at target sites. All the other mutant proteins were also checked for their localization pattern and none of them show any anomaly in their nuclear localization pattern (Supplementary Figure 3B).

#### (ii) O-GlcNAc does not contribute to NuMat association of BEAF 32B

Several studies suggest that a possible mechanism of function of insulator elements may be by tethering chromatin to nuclear architectural components.^8,30,32,33^ NuMat is an essential component of nuclear architecture and BEAF 32 is shown to be a component of NuMat. We wished to find out whether O-GlcNAcylation of the protein plays a role in this association.^30^ NuMat protein fraction was prepared from S2 cells expressing FLAG-tagged wt and T91A mutant BEAF 32B proteins. Nuclear proteins, NuMat proteins, and proteins extracted with DNaseI/2M NaCl were resolved by PAGE and probed with anti-FLAG. T91A mutant protein is resistant to extraction with DNaseI and high salt (Fig. 4B). It remains associated with NuMat in quantitatively similar amounts as wt BEAF 32B protein. Western with a known NuMat protein, Lamin Dm0, was used as positive control. Complete extraction of histones was used as a measure to ascertain the quality of the NuMat preparation. The observation suggests that O-GlcNAcylation of BEAF 32B is not important for its NuMat association.

#### (iii) T91 of BEAF 32B is not a site for phosphorylation

Phosphorylation site of BEAF 32 has not been mapped so far. It is known that serine and threonine residues support phosphorylation as well as O-GlcNAcylation and significant crosstalk occurs between the 2 types of PTMs. Although, results till now suggested that phosphorylation and O-GlcNAcylation in BEAF 32B are not linked, we still wanted to check whether T91 is a phospho-site as well. To confirm this, wt and T91A mutant BEAF 32B proteins were expressed in bacteria. Bacterial extract containing the expressed protein was incubated with S2 cell extract and gamma labeled ^32^P-ATP as phosphate donor. After the reaction, wt and T91A mutant proteins were immuno-precipitated from the bacterial extract with anti BEAF. The precipitated proteins were western blotted with anti BEAF to check the efficiency of pull-down (Fig. 4C, upper panel). After western blotting, the same blot was exposed to phosphor imaging screen to visualize ^32^P-labeled protein. As shown (Fig. 4C lower panel), the wt as well as T91A mutated BEAF 32 were phoshorylated *in vitro*, implying that kinase from S2 cell extract could phosphorylate both wt and T91A mutated protein and T91 was not the site for phosphorylation.

#### (iv) O-GlcNAc does not contribute to the multimerization of BEAF 32B

BEAF 32B is known to form homo- and heteromeric trimer with 32A. We wished to investigate whether its multimerization capacity is impaired if the O-GlcNAc modification is missing. S2 cell lysate (control i.e, untreated S2 cells) and from cells expressing FLAG-tagged wt and T91A mutant BEAF 32B proteins, were treated with the chemical crosslinker DSG (disuccinimidyl glutarate). Cell lysates from crosslinked cells were then resolved by SDS-PAGE and probed with anti BEAF or anti FLAG. The amount of dimer/trimer formed by native BEAF 32 in untreated S2 cells and by exogenously expressed wt and T91A mutant proteins in transfected S2 cells is comparable (Fig. 4D). This indicates that O-GlcNAcylation of BEAF 32B is not important for its functional multimerization.

### T91A mutation does not affect BEAF 32B boundary activity

**Figure 5.**
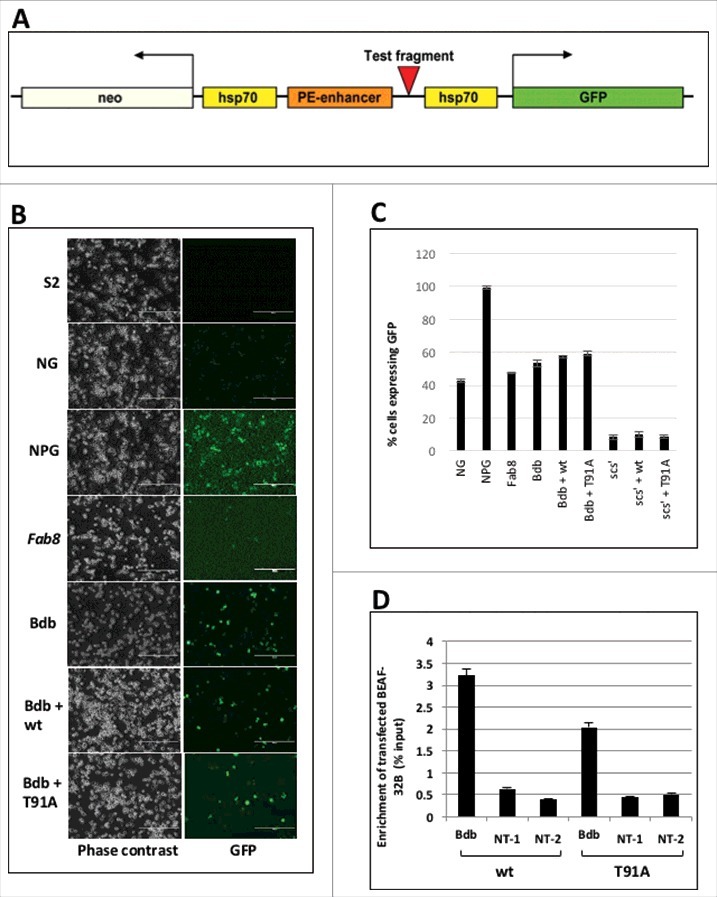
T91A mutant protein has minimal effect on enhancer blocking activity. A. Map of the NPG boundary assay vector. B. T91A mutant does not affect BEAF 32 dependent boundary (*Bdb*) function. Different transformed cell cultures were imaged with fluorescent microscope to view GFP expression. S2 – untransfected S2 cells; NG – Cell line with integrated NG vector that lacks an enhancer; NPG – Cell line with integrated NPG vector; *Fab8* – Cell line with integrated NPG vector with *Fab8* as test element; *Bdb* – Cell line with integrated NPG vector with *Bdb* as test element; *Bdb*+wt – Bdb cell line with exogenously expressed wt protein; *Bdb*+T91A – Bdb cell line with exogenously expressed T91A mutant protein. Scale bar – 200 µM. C. Quantitative estimate of GFP expressing cells from boundary assay cell lines. GFP expressing cells shown in Figure 5B as well as scs' cell line, *scs'*+wt and *scs'*+T91A, were FACS sorted, counted and plotted as % GFP expressing cells. D. Exogenously expressed wt and T91A proteins occupy *Bdb*. Bdb cells expressing FLAG-tagged wt and T91A proteins were cross-linked and immunoprecipitated with anti-FLAG. FLAG-tagged protein occupancy on *Bdb* and 2 other non-target loci (NT-1, NT-2) was estimated by quantitative PCR.

The most studied role of BEAF 32 is in functioning of boundary elements. To test the potential role of O-GlcNAcylation of BEAF 32B in boundary activity, we designed an assay using NPG vector (Neomycin PE enhancer GFP). The vector has 2 reporters, neomycin gene for selection of the stably transfected cells and GFP reporter to assay the enhancer blocking activity (Fig. 5A). The expression of both the reporters are controlled by independent hsp70 promoters which in turn is driven by the twist gene's Proximal Element (PE) enhancer. The PE enhancer was amplified from *Drosophila* genomic DNA using specific primers and the enhancer has been found to work efficiently in S2 cells. The vector has been previously used in a similar S2 cell based enhancer blocker assay.^34^ Five independent transgenic S2 cell lines were generated using i) NG – does not have the enhancer to drive the reporter expression, ii) NPG – is the complete empty vector that has the PE enhancer along with the hsp70 promoter and reporter but lacks any test fragment, iii) Fab8 cell line – has the *Fab8* boundary cloned as positive test fragment between the enhancer and the promoter (iv) NPG vector containing a BEAF 32 dependent boundary (*Bdb*) as test element for boundary activity. The *Bdb* element is a previously characterized BEAF 32 dependent boundary element located in the *Drosophila* bithorax complex and has 7 CGATA sequence in different orientation.^18,34^ In the previous publication, the element is referred to as 3R_422 insulator. (v) NPG vector containing *scs'* boundary element, which is a well characterized BEAF 32 dependent boundary. Cells were selected with neomycin for 6 weeks to obtain stable transformants. After 6 weeks, the cells were transfected with constructs expressing wt and T91A mutant proteins. After 36 hrs of transfection cells were imaged using a fluorescence microscope to check for change in the number of GFP expressing cells. Cells were then sorted using FACS to get a quantitative estimate of GFP expressing cells.

Figure 5C summarizes the results of boundary assay. The NPG cells showed maximum GFP expression. They were the positive control in the experiment and we extrapolated the number of GFP expressing cells from NPG cell line to 100%. Numbers of GFP expressing cells from the other cell lines were compared with the NPG line. In NG cells, where there is no enhancer and only the promoter drives GFP expression, the percentage of cells expressing GFP was 42.6%. In Fab8 cells, where the CTCF dependent boundary abrogates enhancer-promoter interaction the percentage of GFP expressing cells is reduced almost to the level of NG cells (47.56%). This indicated that the assay system was working as the PE enhancer was effectively blocked by a functional *Fab8* boundary element resulting in reduction in the number of cells expressing GFP. In the Bdb cell line, the BEAF 32 dependent boundary reduced the GFP expression to 50%. The level of boundary function remained the same even when wt or T91A mutant BEAF 32B proteins were expressed exogenously. We further used scs' cell line, as the element is a well characterized BEAF 32 dependent boundary. The *scs'* element acts as a very strong boundary, reducing the expression of GFP to almost 10% as compared with NPG lines. This boundary function remained unchanged when wt or T91A mutant BEAF 32B were expressed exogenously. As T91A mutant protein had no effect on boundary function we conclude that O-GlcNAcylation of BEAF 32B is not important for boundary activity (Fig. 5B and 5C). Our finding aligns with earlier study which has shown that boundary activity of BEAF 32 is due to the C-terminal domain of BEAF 32.^35^ In parallel experiments, we tested the other mutant BEAF 32B proteins for their effect on boundary activity. The mutants S196A, S220P, S220A, T190A and S197A had negligible effect on the boundary activity (Supplementary Figure 4).

In the assay described above, wt and T91A mutant BEAF 32B proteins were expressed transiently from pFPc19 vector. The assay was performed 36 hrs after transfection to provide sufficient time for expression of exogenous protein, as well as to allow the cells to undergo at least one replication cycle. We presume that the time provided would have been sufficient to allow the transiently expressed wt or T91A mutant protein to occupy the *Bdb* test loci. However, if that were not the case it would lead to an improper assay. Thus, it was essential to confirm that exogenously expressed proteins were bound to the test DNA element at the time of the assay. As the exogenously expressed proteins had a FLAG-tag, ChIP was done using anti FLAG after 36 hrs of transfection. The precipitated chromatin was checked for the presence of the *Bdb* element using quantitative PCR primers designed against the element (Supplementary Table 2). As negative control 2 primers were designed which amplify BEAF 32 non-target sites (NT-1, NT-2). We saw a 6-fold higher FLAG-tagged protein occupancy at *Bdb* element as compared with its presence at BEAF 32 non-target sites (Fig. 5D). This proved that exogenously expressed wt and T91A mutant BEAF 32B were sitting on the *Bdb* element in the boundary assay and confirm the validity of the assay.

### O-GlcNAcylation of BEAF 32B correlates with H3K4me3 marks

**Figure 6.**
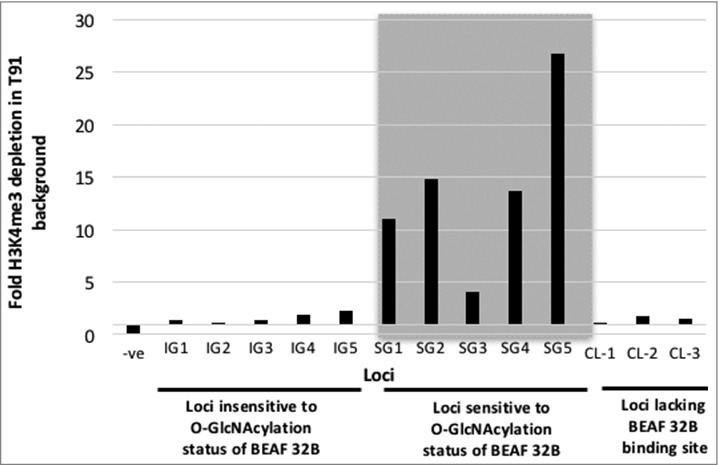
T91A mutation correlates with reduced H3K4me3 on promoters of associated genes. Enrichment of H3K4me3 was calculated for each locus in the presence of exogenously expressed wt or T91A BEAF 32 protein. H3K4me3 ChIP followed by quantitative PCR was used for this purpose. To obtain the fold depletion, the H3K4me3 enrichment in wt background was divided by that in T91A background. Locus lacking H3K4me3 was used as ‘–ve’ control; IG1–5 are loci that were minimally affected by O-GlcNAcylation status of BEAF 32B; SG1–5 are loci that were severely affected by O-GlcNAcylation status of BEAF 32B; CL-1–CL-3 are loci enriched for H3K4me3 independent of BEAF 32B.

It is known that BEAF 32 binds to TSS of transcriptionally active genes and maintains the associated promoter regions in an environment that facilitates high level of transcription. Many of these associated genes have cell cycle, development and differentiation related function. Such active promoters are also enriched with modified histone H3K4me3. It has also been reported that absence of BEAF 32 leads to decrease in transcription of linked genes.^20^ We wanted to explore the role of O-GlcNAcylation of BEAF 32B in deposition of H3K4me3 marks at the promoters where it binds. For this we queried 14 different loci that were chosen based on modENCODE data for H3K4me3 enrichment and ChIP-chip data for BEAF 32B occupancy.^18^ The sites were chosen such that both H3K4me3 occupancy and BEAF 32B binding had a high score in their respective binding studies. We performed H3K4me3 ChIP and confirmed by quantitative PCR that the chosen negative control locus lacked H3K4me3, whereas the other 13 showed enrichment of variable levels. Of the 13, the 3 control loci (CL-1 to CL-3) had only H3K4me3 enrichment and no BEAF 32B binding. As the H3K4me3 deposition at these control regions is independent of BEAF 32, O-GlcNAcylation status of the protein is not expected to influence the modified histone levels. Rest of the 10 loci were bound to BEAF 32B as well as were enriched with H3K4me3. All these loci were in TSS of genes. S2 cells transfected with wt or T91A mutant BEAF 32B proteins were grown for 36 hours and H3K4me3 ChIP was performed with them. Fold enrichment of the 14 test loci as detailed above, was estimated in the immuno-precipitated DNA by quantitative PCR using primers listed in Supplementary Table 3. Enrichment at each locus in wt background was compared with its enrichment in T91A background (Supplementary Figure 5). As expected the negative control locus was devoid of H3K4me3 mark. At control loci (CL1-CL3), the levels of H3K4me3 was not effected by exogenous expression of wt or T91A. However, the BEAF 32B bound loci (IG1–5 and SG1–5) were effected, where exogenous expression of T91A lead to a depletion in H3K4me4 level. To obtain the fold depletion, enrichment value obtained from cells transfected with wt BEAF 32B was divided by the enrichment value obtained from cells transfected with T91A mutant BEAF 32B and plotted in a graph (Fig. 6). Based on the fold depletion value, the BEAF 32B bound loci could be grouped in 2 categories, namely, loci showing insignificant depletion and loci showing significant depletion of H3K4me3. We labeled them as loci insensitive to BEAF 32B O-GlcNAcylation status (IG1–5) and loci sensitive to the PTM status (SG1–5. Interestingly, the 5 loci that showed significant H3K4me3 depletion were TSS of genes related to cell cycle, development and differentiation. TSS of genes associated with other functions were not affected. The GO annotations for the genes were obtained from FlyBase (http://flybase.org/) and are listed in Table 1. Based on our experiment we conclude that O-GlcNAc modification of BEAF 32 relates to deposition of H3K4me3 on TSS of associated genes and this effect is more prominent on cell cycle, development and differentiation related genes.

### O-GlcNAcylation of BEAF 32B is linked to transcription of associated genes

**Figure 7.**
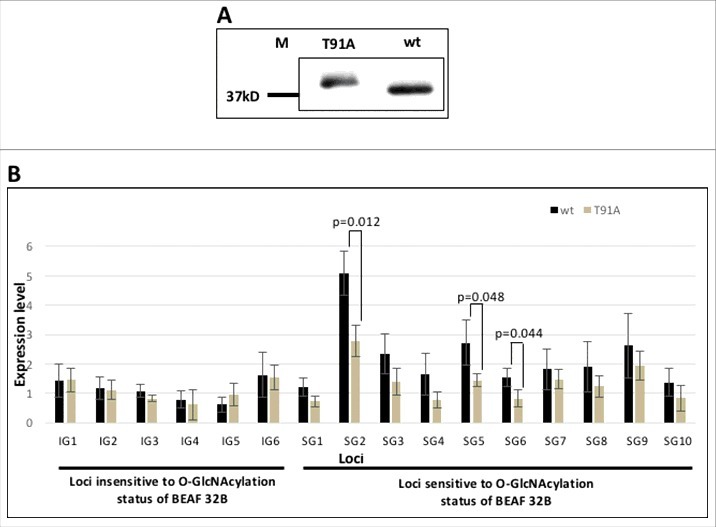
Lack of O-GlcNAcylation on BEAF 32B reduces the expression level of a subset of associated genes. A. FLAG tagged wt and T91A BEAF 32B was transfected into S2 cells and protein expression was checked by western with anti-FLAG. B. cDNA was prepared from wt and T91A transfected cells and expression level of genes was checked by quantitative PCR. Although genes associated with loci IG1–6 did not show significant change, those associated with loci SG1–10 show decrease in expression in T91A background as compared with the wt background (*P < 0.05).

Previous result showed that BEAF 32 O-GlcNAcylation is related to H3K4me3 level of associated genes. We wanted to check whether the level of activation mark correlates with the transcription status of linked genes. To increase the confidence, we included few more genes (IG6 and SG6–10) which are involved in similar biologic processes and have BEAF 32B binding at their TSS, for checking the expression levels under wt and T91A BEAF 32B protein expression background (Table 1). We expressed wt and T91A BEAF 32B proteins in S2 cells and harvested the cells after 36 hrs. Expression level of the exogenously expressed proteins in transfected cells was confirmed by western with anti-FLAG (Fig. 7A). Total RNA was prepared from these cells, from which mRNA was reverse transcribed to yield cDNA. Using quantitative PCR the transcript level of the queried genes was checked (Primers listed in Supplementary Table 4). Act5C transcripts, which is constitutively expressed at a similar level in both transfected and un-transfected cells, was used as the reference for normalization. Expression level of target genes in wt and T91A mutant BEAF 32B expression background was compared against the expression level of the same genes in un-transfected S2 cells (Fig. 7B). We observed that genes linked with loci IG1 to IG6 do not show significant difference in their level of expression in wt and T91A background, but genes linked with loci SG1 to SG10 show an apparent decrease in expression when transfected with T91A BEAF 32 as compared with wt BEAF 32. Although the number of loci queried is small, the trend clearly shows that transcription of SG1 to SG10 reduces in presence of T91A. Specifically SG2, 5 and 6 show a significant decrease in expression in T91A transfected cells. Our results suggest that lack of O-GlcNAcylation of BEAF 32B reduces expression level of a subset of its target genes that are mostly associated with cell cycle, development and differentiation related function.

### BEAF 32 is O-GlcNAcylated and phosphorylated in dividing/differentiating tissues

**Figure 8.**
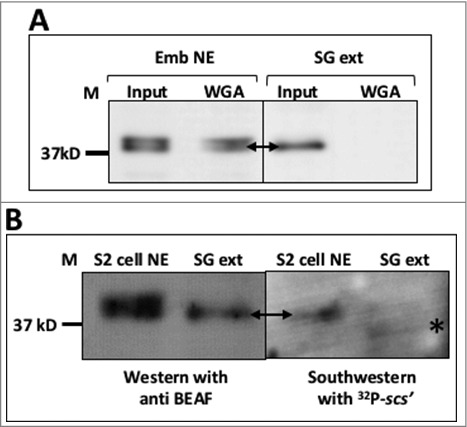
BEAF 32 O-GlcNAcylated and phosphorylated in differentiating tissues. A. O-GlcNAcylated BEAF 32 was affinity purified by WGA agarose beads from embryo nuclear extract (Emb NE) and salivary gland extract (SG Extract). Blot was probed with anti BEAF. Only the lower dephosphorylated isoform of BEAF 32 is present in SG extract. Affinity purified fraction in ‘WGA’ lanes show that only embryonic BEAF 32 is O-GlcNAcylated. B. S2 cell NE and SG extract were resolved on 12% SDS-PAGE and western blotted with anti BEAF. Southwestern hybridization was performed on the same blot with ^32^P-labeled *scs'* DNA to show that the lower dephosphorylated isoform of BEAF 32 binds to DNA. M – molecular weight marker.

All the above experiments were done in S2 cells which represents dividing cells of *Drosophila melanogaster* embryonic lineage. These cells are not ideal for study of development/differentiation related mechanisms. To explore the role of O-GlcNAcylation in an organism, we used embryos as a developing/differentiating tissue where cells were actively dividing as well as differentiating. Salivary glands of third instar larvae was used as an example of fully differentiated tissue where cells were no longer dividing. Embryos (0–16 hrs) were collected and salivary glands from third instar larvae were dissected out. NE was made from them as described in materials and methods. Proteins from the NE was used for pull-down by WGA agarose beads. Western blot analysis of the pulled down proteins with anti BEAF showed that BEAF 32 although present in both tissue types, is O-GlcNAcylated only in embryo and not in larval salivary gland (Fig. 8A). Interestingly, only the dephosphorylated isoform of BEAF 32 exists in salivary glands (Fig. 8A & B). The dephosphorylated isoform binds to target DNA as well (Fig. 8B). However, this interesting observation warrants that tissue-specific phosphorylation of BEAF 32 needs to be explored in further detail. Our study shows that, O-GlcNAcylation as well as phosphorylation of BEAF 32 is present in dividing and/or differentiating tissue but the PTMs are absent in an already differentiated tissue. Further experiments using more tissue types need to be done to confirm the interesting observation, however our result suggests a co-relation between PTMs of BEAF 32 and cell division/differentiation.

### Discussion

BEAF 32 was discovered as a protein which can bind to DNA and prevent enhancer-promoter interaction.^17^ However, genome wide binding studies have revealed that the role of the protein is not limited to boundary activity. DNA sequences bound by BEAF 32 when tested in transgenic context (*scs'* being most prominent of them) function as boundaries/insulators, but in the genomic context the protein appears to play a wider role.^19,34^ Interestingly, more than 85% of BEAF 32 peaks are located within 300 bp of TSS of genes. Many of these genes are related to cell cycle, chromosome organization/segregation, development and differentiation.^18,20^ Accordingly, depletion of BEAF 32 leads to cell cycle and chromosome segregation defects. A significant percent (∼85%) of these genes bound by BEAF 32 at their TSS, overlap with the list of housekeeping genes in *Drosophila*.^36^ Interestingly, actively transcribing housekeeping genes are enriched at TAD boundaries.^12^ In majority of the instances, BEAF 32 associated genes are closely spaced head-to-head gene pairs. BEAF 32 bound DNA element separates these genes that display differential pattern of expression and depletion of the protein affects expression levels of one of the gene of the gene pair.^5^ Even at the *scs'* element at the 87A7 hsp70 locus, BEAF 32 binds in the promoter region of head-to-head gene pair of *CG3281* and *aurora*.^37^ These genes do not respond to heat shock, are not coordinately regulated and *aurora* is a cell cycle related kinase.

Studies so far have shown that BEAF 32 relates to several epigenetic processes in the nucleus. The protein along with its co-factors [CP190/Mod(mdg4)], favors long range interactions and establishment of TADs.^38^ It is also known that BEAF 32 alters the chromatin landscape at the TSS of neighboring genes. Its binding sites overlap with active histone marks like H3K4me3 and H3K36me3.^18,37^ Recent studies have shown that the protein interacts with an essential histone methyl transferase, dMes-4, to organize nucleosomes downstream of active promoters.^23,24^

The overall emerging picture thus indicates that BEAF 32 can insulate a pair of associated gene promoters from improper regulation, aid in active transcription of one of the associated gene and make long range contacts to help organization of TADs. Understandably, it carries out these activities by interacting with different co-factors. However, properties of the protein that allow it to interact with different partners is not yet elucidated. BEAF 32 is also known to have PTMs, that have remained largely uncharacterised.^16,17,30^ As PTMs widen the scope of function of a protein when compared with what is dictated by its sequence, we wished to investigate the role of PTMs in BEAF 32 functionality.

**Figure 9.**
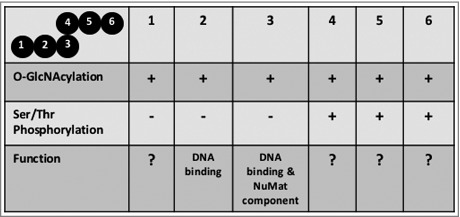
**PTM variants of BEAF 32 and their functions**. Top left cell represents the PTM variants of BEAF 32 graphically. ‘+’ indicates the presence and ‘−’ absence of the respective PTM.

Biochemical studies done here show that BEAF 32 has multiple post translationally modified variants. These variants have different pI and molecular weight and carry out different functions, depending upon the PTM they carry (Fig. 9). Our studies on phosphorylation status of BEAF 32 show that phosphorylated as well as dephosphorylated isoforms of the protein exist in the nucleus. However, only the dephosphorylated isoform binds to DNA and is enriched in NuMat. It is known that reversible phosphorylation can directly regulate distinct aspects of transcription factor function, including sub-cellular localization, conformation, protein-protein interactions and DNA binding. Phosphorylation of residues in DNA-binding domain inhibits the binding of transcription factor to target sites.^39,40^ In such situations dephosphorylation of the protein is an important functional determinant. As we observe that dephosphorylated BEAF 32 binds to DNA and localizes to NuMat, removal of phospho group from the protein might be a critical determinant for its activity. However, the phosphorylated variant also exists inside the nucleus indicating that either phosphorylated BEAF 32 has an additional role in the nucleus or it undergoes rapid dephosphorylation for activation in the nucleus. The precise role of phosphorylation on BEAF 32 can be addressed in a much better way once the residue(s) undergoing the modification have been mapped in order to mutate it for further studies.

We have studied O-GlcNAcylation of BEAF 32 in detail. Unlike the complex glycans embellishing the cell surface, protein O-GlcNAcylation is a reversible single sugar modification on the hydroxyl group of Ser/Thr residues.^41,42^ In *Drosophila*, many nuclear proteins are O-GlcNAcylated as visualised by polytene chromosome staining with fluorescent WGA.^43^ It is similar to protein phosphorylation in terms of stoichiometry, localization, and cycling. O-GlcNAcylation is under a unique level of metabolic control and has physiologic significance as a nutrient sensor.^44^ O-GlcNAc transferase (OGT) is the only enzyme known to be responsible for carrying out this modification that is an essential and highly conserved protein.^45^ The sugar donor substrate that drives OGT depends on the levels of glucose and this provides a biochemical sensor that enables cells to adapt to the presence of low, normal or high glucose levels.^46,47^ Along with nutrient levels, other cues like cellular stress, tissue injury and levels of insulin also lead to increase in the amount of O-GlcNAcylated proteins.^48,49^

We have mapped O-GlcNAcylation of BEAF 32 to the 91^st^ residue of the protein which is a threonine. T91 residue is conserved in one of the clade (the *melanogaster* group of *Sophophora* subgenus) of the *Drosophila* genus (Fig. 2A). Given its importance we expect some other conserved residue to be supporting O-GlcNAcylation of BEAF 32 in the other clade of *Drosophila* genus. Mutating the T91 residue to alanine gave us much insight in relating the role of O-GlcNAcylation of BEAF 32 to its function. Residue T91 is neither a part of the N-terminal DNA binding region nor C-terminal protein-protein interaction region of BEAF 32. It lies in the middle region of BEAF 32 distinctly away from the region reported to be responsible for its NuMat association. This suggests that O-GlcNAcylation at T91 may not have any role in DNA binding activity or boundary activity of BEAF 32 as these activities require N-terminal and C-terminal domains respectively.^30^ Observations in our subsequent experiments show that lack of O-GlcNAcylation at T91 does not affect multimerization, nuclear localization and NuMat binding ability of BEAF 32. Also, using a cell-based transgenic assay, we show that absence of O-GlcNAcylation has a negligible effect on the capability of BEAF 32 to act as a boundary protein. This assay has the limitation that the endogenous BEAF 32 is also present which can dilute the effect of T91A mutant. However, we ensured that expression level of mutant protein is comparable to the level of endogenous BEAF 32 and sufficient time is provided during the assay to allow the mutant to occupy the test locus. As threonine residues are often known to undergo phosphorylation and O-GlcNAcylation in a mutually exclusive manner,^50^ we confirmed that T91A mutant still gets phosphorylated. Thus, T91 in BEAF 32 is not a phospho-site and O-GlcNAcylation and phosphorylation modifications of the protein are independent of each other.

Several examples of O-GlcNAc modification of transcription factors that eventually lead to regulations of transcription are known.^51-54^ Studies in mammalian cells has highlighted a situation where O-GlcNAcylation of protein HCF1 can influence the deposition of H3K4me3 mark and lead to active transcription.^55^ To explore a possibly similar situation, we checked the correlation between O-GlcNAcylation of BEAF 32B and occurrence of H3K4me3 mark by ChIP assay. We observed that abolition of O-GlcNAc modification of BEAF 32B affected H3K4me3 occupancy at TSS bound by BEAF 32B. Interestingly the affected genes were mostly involved in cell cycle, development and differentiation. This observation is highly intriguing as it implies that O-GlcNAcylation of BEAF 32 is related to regulation of a specific subset of its target genes. This also indicates toward additional layers of regulation that remain to be explored.

loaded off-line onto a nanospray tapered capillaryThis interesting observation is supported by the result that BEAF 32B is O-GlcNAcylated as well as phosphorylated in dividing cells (S2 cells) and differentiating tissue (0–16 hr embryos) but not in terminally differentiated tissue (salivary glands). This also suggests that PTMs of BEAF 32 acts as a sensor to create an active chromatin landscape conducive to the transcription of only a subset of linked genes possibly in response to environmental factors such as glucose availability or nutrient/energy status. Knowledge of PTMs of BEAF 32 in S2 cells may prove to be an useful resource for such a line of investigation, as glucose levels can be easily manipulated under culture conditions.

In conclusion, we find that BEAF 32 is substrate for multiple PTMs including phosphorylation and O-GlcNAcylation, which results in several variants of the protein. There is a clear link between its O-GlcNAcylation and deposition of H3K4me3 marks at TSS of associated genes. The loss of the activation mark in absence of O-GlcNAcylation of BEAF 32B, reduces the expression of level the linked genes to varying degrees. This reversible modification may confer different regulatory properties to the protein depending on the context and in response to variable environmental cues. Our study highlights the capability of a PTM to diversify the function of a protein.

## Materials and methods

### Plasmids, protein expression and site directed mutagenesis

The parent plasmid pFPc19 was obtained from R.E Kingston. It is a pCaSpeR4-based vector carrying a part of the polycomb promoter (845 bp) followed by a FLAG tag of 8 amino acids (MDYKDDDK) at the N-ter of the polycomb gene coding sequence. For our experiments, we replaced the polycomb gene with BEAF 32B coding sequence and various mutants of BEAF 32B. Polycomb promoter drives the expression of FLAG-tagged proteins in S2 cells.

BL21 (DE3) cells transformed with pET-28a carrying the coding sequences of BEAF 32B were induced to express the protein in bacteria. Expression was induced with 2mM IPTG for 4 hours. Bacterial cell lysates were analyzed by western blotting.

Site directed mutants (SDM) in BEAF 32 were generated using the necessary primers and DpnI enzyme. Primers were designed so as to generate necessary base changes in the mutants. BEAF 32B coding sequence in pBSK was used as template to amplify the mutant fragments. KOD DNA polymerase (Novagen) with proof reading activity was used for PCR amplification. The PCR product was digested with DpnI enzyme for 2 hrs at 37°C. DpnI enzyme cleaves off the initial template DNA of bacterial origin as it has the property to cleave methylated DNA. The digestion reaction was cleaned up using column and the DNA was transformed into DH5α cells. The linearized mutated plasmid gets repaired in bacteria. Bacterial colonies were screened for transformants and the mutant plasmid was amplified. Finally, the mutant BEAF 32B coding sequences were excised from pBSK and cloned into pFPc vector.

### S2 cell NE preparation

One million S2 cells grown in Schneider media were collected and washed twice with PBS at 900 X g at 4°C for 5 mins. The cells were re-suspended in 300 µl of NIB [3.75 mM Tris (pH 7.4), 20 mM KCl, 0.5 mM EDTA, 0.125 mM Spermidine, 0.05 mM Spermine, 0.1 mM PMSF] and kept at room temperature for 5 min. After incubation, they were passed 10 times through a 22 gauge needle to rupture the cells and release the nuclei. The nuclei were collected by spinning at 3000 X g for 5 min. Using NIB, nuclei were washed twice by spinning at 3000 X g for 5 mins. The washed nuclei were resuspended in 300 µl of Nuclear extraction buffer (NEB)-20 [10mM HEPES (pH7.6), 20 mM KCl, 3 mM MgCl_2_, 1 mM EDTA, 10% Glycerol and Protease inhibitor]. Equal volume of NEB-700 [10 mM HEPES (pH7.6), 700 mM KCl, 3 mM MgCl_2_, 1 mM EDTA, 10% Glycerol and Protease inhibitor] was added to it. The nuclei were extracted for 2 hrs in cold room with gentle rotation using a rotator. This extract was centrifuged for 1 hr at 42000 rpm and 4°C using a table top ultra-centrifuge to remove all the debris. The NE was snap frozen in liquid nitrogen and stored at −80°C.

### Phosphatase treatment of NE

NE as prepared above was treated with Shrimp Alkaline phosphatase (SAP – NEB) in the buffer provided by manufacturer for an hour at 37°C. SDS gel loading dye was added to the treated extract and boiled for 3 min. The phosphatase treated extract was analyzed by 1D/2D western blot.

### O-GlcNAc site mapping of BEAF by LC-MS/MS

Tryptic peptides were resuspended with 19.5 μL of mobile phase A (0.1% formic acid, FA, in water) and 0.5 μL of mobile phase B (80% acetonitrile, ACN, and 0.1% formic acid in water) and filtered with 0.2 μm filters (Nanosep, PALL). The sample was loaded off-line onto a nanospray tapered capillary column/emitter (360 • 75 • 15 μm, PicoFrit, New Objective) self-packed with C18 reverse-phase (RP) resin (10.5 cm, Waters) in a nitrogen pressure injection cell for 10 min at 1000 psi (∼5 μL load) and then separated via a 160 min linear gradient of increasing mobile phase B at a flow rate of ∼200 nL/min directly into the mass spectrometer. LC-MS/MS analysis was performed on a LTQ Orbitrap XL mass spectrometer (ThermoFisher, San Jose, CA) equipped with a nanospray ion source. A full FTMS (Fourier transform mass spectrometry) spectrum at 30,000 resolution was collected at 300–2000 m/z followed by 5 data dependent MS/MS spectra of ITMS (Ion trap mass spectrometry) in the most intense ion peaks from parent mass list following CID (36% normalized collision energy). The parent mass width was set up ± 20.0 ppm. To obtain the parent mass list, the protein sequences was theoretically digested by trypsin allowing to one internal miss cleavage. The masses of theoretical tryptic peptides were allowed for dynamic modifications with the masses of oxidized methionine, alkylated cysteine, and maximum 3 of GlcNAc (15.9949, 57.0215 and 203.0794 Da) respectively and then calculated with up to quintuply charge states. The masses were selected between 300–2000 m/z at each charge state for the parent mass list.

After the O-GlcNAc peptide was identified by MS/MS, the mass of O-GlcNAc modified peptide was trapped to acquire the FT MS spectrum of MS/MS without collision energy by LC-MS/MS.

The resulting data was searched against a BEAF 32 sequence database containing the common contaminants database (ThermoFisher) using the TurboSequest algorithm (BioWorks 3.3.1 SP1, Thermo Fisher Scientific, Inc.). Spectra with a threshold of 15 ions, a TIC of 1 • 103, and a mass range of [MH]+ = 600–5000 m/z were searched. The SEQUEST parameters were set to allow 30.0 ppm of precursor ion mass tolerance and 0.5 Da of fragment ion tolerance with monoisotopic mass. Only fully tryptic peptides were allowed with up to one missed internal cleavage sites. Dynamic mass increases of 15.9949, 57.0215, and 203.0794 Da were allowed for oxidized methionine, alkylated cysteine, and O-GlcNAcylated serine/threonine respectively. The results of the SEQUEST search were filtered at ≥ 0.60 Final Score (Sf) but all O-GlcNAc modified peptide fragmentation was manually validated without filtering.

### BEAF 32B Sequence alignment from different *Drosophila* species

The *Drosophila melanogaster* BEAF 32 gene sequence was used to find its homologous regions in other *Drosophila* species. The homologous regions were extended 5Kb upstream/downstream and subjected for gene prediction using Genescan tool. The predicted peptides were assigned as BEAF 32A or 32B based on its homology to *Drosophila melanogaster* isoforms. For many species the gene predictions could be validated based on available EST sequences. The multiple sequence alignment for each isoforms was done using ClustalW & ClustalX. The phylogenetic analysis was performed using MEGA software.

### WGA pull down from NE

Total protein content in the NE was quantitated and ∼100 µg protein was diluted in PBST (1XPBS with 0.01% Tween-20) to make up the final volume to 200 µl. WGA beads (50 µl) (Thermo Scientific™ Pierce™) were added to the diluted NE for each pull down. Pull down was done by incubating the NE with WGA beads on a rotator with slow rotation of 15 or 16 rpm overnight in cold room. The beads were then washed 3 times with PBST and finally suspended in SDS gel loading dye for western blot analysis.

### 1D/2D gel electrophoresis, western and southwestern blotting

NE was analyzed by 1D/2D SDS-PAGE, transferred to PVDF membrane and probed with antibodies to BEAF (DSHB, dilution 1:1000) or FLAG (Sigma, dilution 1:5000). The blots were developed using chemiluminescence kit from Perkin Elmer as per the manufacturer's protocol. 2D gel electrophoresis was performed using the Protean II electrophoresis setup (Bio-Rad). NE (100 µg) was resolved on the Ready Strips IPG strip from Bio-Rad (3–10 NL). The 2D run was performed on a 12% SDS-polyacrylamide slab gel. 2D gels were also processed for western blotting as described above.

For southwestern blotting, proteins resolved on 2D-gel were transferred to PVDF membrane. Proteins on membrane were denatured with 8M GnHCl for 10 min and slowly re-natured by diluting the GnHCl in a stepwise manner. The membrane was blocked in 5% BSA and hybridized to ^32^P-labeled scs' probe DNA overnight. The blot was washed stringently and exposed to phosphor imaging screen to obtain the image.

### Immunofluorescence and confocal microscopy

Control/transfected S2 cells were grown on coverslip for 24 hours. Cells were fixed with 4% formaldehyde in PBS containing 0.1% Triton X-100 for 5 min. After extensive washing, anti FLAG/BEAF (1:100 in PBST) was added and incubation was performed at 4°C overnight. After secondary antibody incubation and washings, the coverslips were mounted with mounting media containing DAPI (Vectasheild, Vector laboratories). Slides were imaged using appropriate excitation wavelength of laser light on a Leica SP8 confocal microscope. Images were processed using Leica Application Suite Advanced Fluorescence (LAS AF) software. Pearson's correlation coefficient value as a measure of co-localization was also calculated using the software. Any value of Pearson's correlation coefficient from 0.6 to 0.9 is considered as significant co-localization.

### NuMat preparation

NuMat was prepared from S2 cells expressing wt/mutant BEAF 32B protein, according to protocol published earlier.^30^ The nuclear, NuMat and extracted proteins were analyzed by western blotting.

### *In vitro* phosphorylation of protein

S2 cells were lysed using lysis buffer (20 mM Tris (pH7.9), 1 mM MgCl_2_, 1 mM EDTA, 0.5% Triton X-100, 10% Glycerol, Protease inhibitor). Lysate was spun at top speed in micro centrifuge to pellet debris. Protein extract was frozen in aliquots at −80°C. Bacterial extract (10 µg – that had wt or T91A mutant protein expressed) was incubated with 2 µg of S2 cell extract in a 50 µl reaction buffer [10 mM Tris-HCl (pH 7.5), 10 mM NaCl, 1 mM MgCl_2_, 0.1 mM EDTA, 1 mM DTT, 0.01% Triton X-100, 10 mM Na_3_VO_4_ (for phosphatase inhibition) and 10 µCi of γ-^32^P ATP as phosphate donor]. The reaction was performed for 30 min at 30°C. In parallel a control reaction was performed were no γ-^32^P ATP was added. After the incubation, the reactions were split into 2 (25 µl each) and diluted again to 50 µl with pull down buffer (1X PBS with 0.01% Tween-20). To one half anti BEAF was added and the other was kept as control without antibody. After immunoprecipitation, the pulled down proteins were resolved on SDS-PAGE, transferred to PVDF and probed with anti BEAF. After the western the PVDF membrane was exposed to phosphor imaging screen for 24 hours.

### DSG crosslinking

DSG (disuccinimidyl glutarate) was used to cross-link the proteins with their native interactions to visualize protein multimers in SDS-PAGE. Transfected S2 cells were grown for 24 hrs. Cells were then collected, washed with PBS and re-suspended in crosslinking buffer (1X PBS, 0.1M NaCl, 0.05M DTT, 1mM PMSF, 0.1% Triton X-100, substituted with protease inhibitor). Cells were lysed by passing through a 22 gauge needle 10 times and the lysate was centrifuged at 14,000 rpm for 30 min at 4°C to remove debris. The lysate was treated with 2 mM DSG (25 mM stock in DMSO) at room temperature for 20 min. Crosslinking was stopped by precipitating proteins with 20% TCA. The precipitated proteins were analyzed by SDS-PAGE and western blotting.

### Boundary assay

Selecting S2 cells transfected with NPG vector for neomycin resistance, generated stable cell line expressing eGFP driven by HSP70 promoter. This cell line formed our control cell line for boundary assay. Known boundary elements, *Fab8, Bdb* or *scs'* were cloned in between PE enhancer and HSP 70 promoter.^34^ The extent of repression was estimated by counting the reduction in number of GFP expressing cells. Wild type protein and its mutants were transfected in the *Bdb* containing stable cell line. After 36 hrs of transfection, the cells were collected and subjected to FACS sorting using MoFlo cell sorter. The increase or decrease in number of GFP positive cells was plotted in a graph. To confirm that the FLAG-tagged protein is sitting on the target transgenic loci ChIP with anti FLAG (Sigma) was performed using Millipore's Chromatin Immunoprecipitation (ChIP) Assay Kit (Catalog # 17–295). Briefly, 1 × 10^6^ Bdb cells were transfected and grown for 36 hrs. The cells were then fixed with 4% Formaldehyde for 10 min at room temperature. The cells were re-suspended in 200µl of ChIP lysis buffer provided with the kit and manufacturer protocol was followed further. Sonication was done using Diagenode Bioruptor® Pico (8 cycles, with 30 sec on and 30 sec off at 4°C). Quantitative PCR for enrichment of *Bdb* fragment in ChIP-DNA was done using ViiA™ 7 Real-Time PCR System from Life Technologies with Power SYBR® Green qPCR Master Mix. Enrichment in the ChIP DNA was determined as percentage Input, where Input DNA represents an aliquot of the same crosslinked and sheared chromatin used for ChIP. Two non-target loci were also quantitated for comparison (Supplementary Table 2).

### H3K4me ChIP and quantitative PCR

All the primers used in this study were designed using Primer3 (http://bioinfo.ut.ee/primer3–0.4.0/) and based on *D. Melanogaster* assembly Apr. 2006 (BDGP R5/DM3).

Millipore's ChIP Kit was used for performing ChIP with H3K4me3 antibody (Abcam) as described above. S2 cells transfected with wt and T91A were used. The list of primers used for quantitative PCR is given in Supplementary Table 3. To obtain the fold depletion, enrichment value obtained from cells transfected with wt BEAF 32B was divided by the enrichment value obtained from cells transfected with T91A mutant BEAF 32B.

### cDNA preparation and gene expression analysis

One million cells transfected with wt and T91A mutant BEAF 32B, individually, were harvested after 36 hrs. The cells were re-suspended in 1 ml of TRIzol® reagent (Thermo Fisher Scientific) and total RNA was isolated from them. Total RNA (10 µg) was treated with RNase free DNaseI (Ambion®'s) for 15 min. DNase free RNA (5 µg) was reverse transcribed using SuperScript® III Reverse Transcriptase (ThermoFisher scientific) and oligo dT primers according to the manufacturers protocol to obtain cDNA. Quantitative real-time PCR was performed in triplicate by using SYBR Green PCR Master Mix Detection system (Applied Biosystems). mRNA level of the genes under study were normalized by the mRNA level of the housekeeping gene Act5c. Relative gene expression analysis (ΔΔCt method) was performed for all the queried genes. Sequence of primers used in the process are given in Supplementary table 4. Test for the level of significance of the data obtained was done using student's t-test. For significant differences P-value for pair wise comparison (wt/T91A) was calculated using the 2-tailed t-test. P value < 0.05 was considered significant.

### Fly tissue extract preparation

Salivary gland from third instar larvae (20 pairs) were dissected in ice cold PBS. Tissue was homogenized using a hand-held homogenizer in 200 µl of RIPA buffer (150 mM sodium chloride, 1.0% NP-40, 0.5% sodium deoxycholate, 50 mM Tris, pH 8.0, 1X cOmplete™ EDTA-free Protease Inhibitor). The tissue extract thus prepared was stored in −80°C ultra freezer. Nuclei from fly embryos was prepared according to the published protocol for NuMat preparation. NE from these nuclei was prepared in the same way as was done for preparation of S2 cell NE.

## Supplementary Material

Supplementary Files
