# Supplementary material for: O-GlcNAcylation of boundary element associated factor (BEAF 32) in Drosophila melanogaster correlates with active histone marks at the promoters of its target genes
Source: Nucleus. 2017 Sep 14;9(1):65–86. doi: 10.1080/19491034.2017.1367887 (PMC5973196; doi:10.1080/19491034.2017.1367887)
Supplement: Supplementary Files [file kncl-09-01-1367887-s001.zip › Supplementary Figures.pdf]

Supplementary Figure 1 –

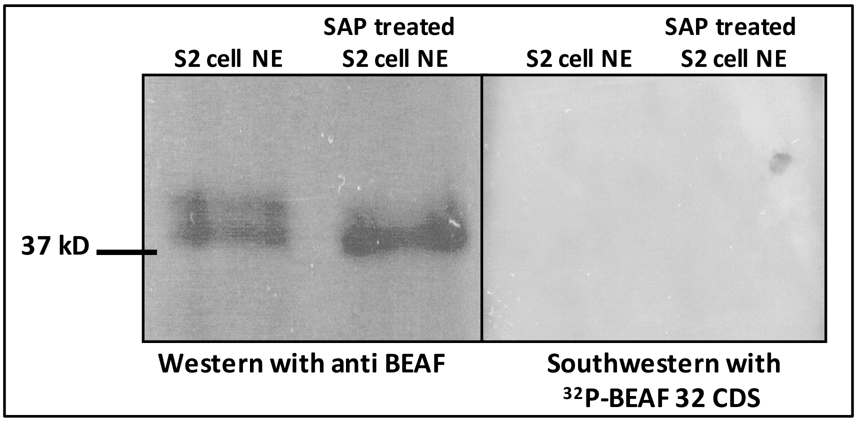

Untreated and SAP treated S2 cell NE were resolved on a 12% SDS-PAGE. The protein blots were probed with anti BEAF antibody. The same blots were then probed with ^{32}P-labeled BEAF 32 protein coding sequence (non-specific DNA).

Supplementary Figure 2 –

BEAF 32B is O-GlcNAcylated at T91

A

MPKGR**VSNVWQHYDINEECENFAICRYCGNNISRGGMASNLKGNNTTN**

LWTHLRHK**HRDEVLVSPVQQRPQARMIPIIK**K**DKTVR**ITK**A^{89}KT$_{g}^{91}$LR^{93}E**

PLR^{97}VAK**PKIESNVANYLGEAGALPQKWEEYEQNDEISEDIK**DIIYSEDPLC

YSPIHVMDDEGLDQPEKQVTVLTHSTSPAGGSSR**PIGVGSGVQATVVASTS**

SSSSSAKQLKNNLETSIERLTAVSEQLSYIIQQNHEELTKDDDYYFALSLVPA

MR**HLSLSRKMYVRSKIQDILFKESEDSTLAKDE**

K A K T L R E

K T L R E P L R V

BEAF 32B sequence showing peptides identified (in blue) and O-GlcNAc site (in red) by LC-MS/MS using parent mass list method.

B

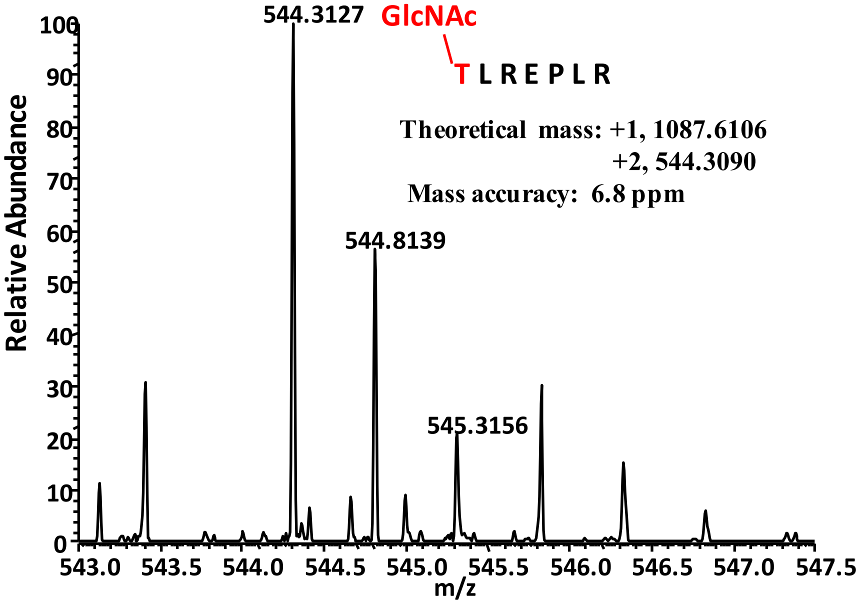

Spectrum showing O-GlcNAc site identified by the Fourier Transform Mass Spectrometer (FT MS) and MS/MS without collision energy.

C

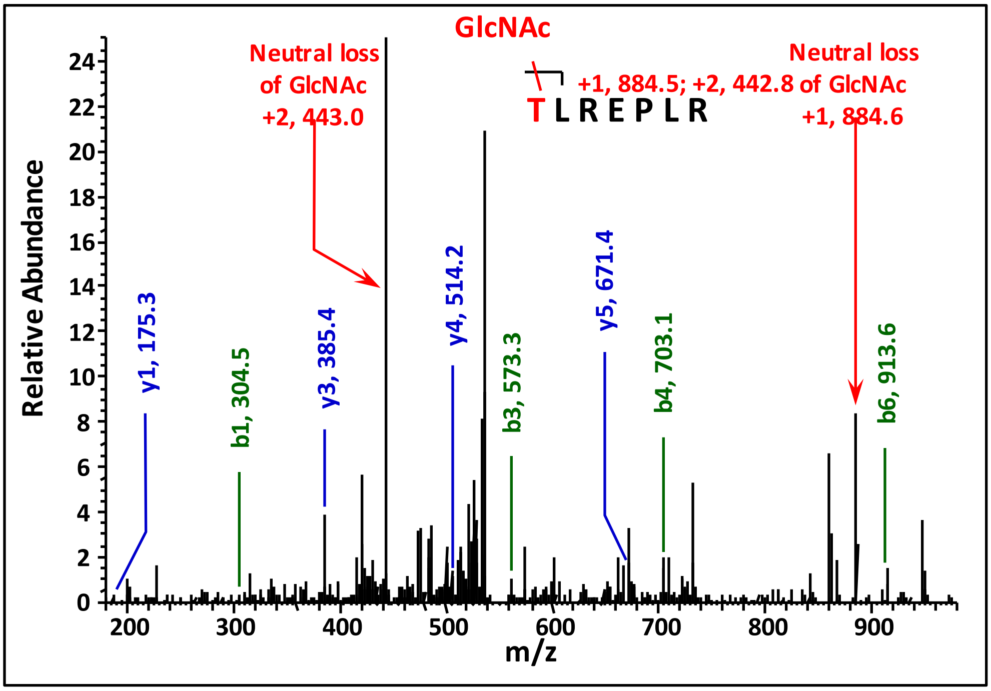

Spectrum showing fragmentation of the O-GlcNAc modified peptide between Thr_g^{91} and Arg^{97} (TgLREPLR).

D

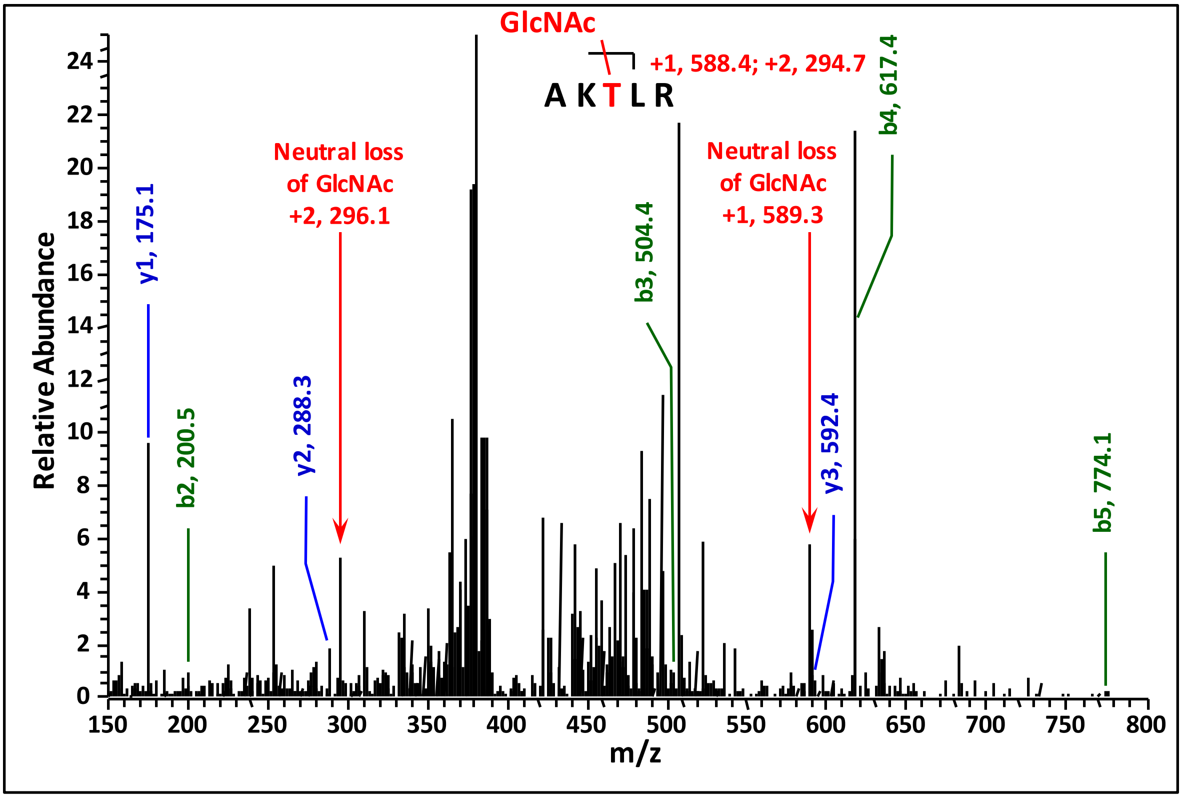

Spectrum showing fragmentation of the O-GlcNAc modified peptide between Ala^{89} and Arg^{93} (AKTgLR).

Supplementary Figure 3A –

Exogenously expressed wt and T91A BEAF 32B co-localize with native BEAF 32

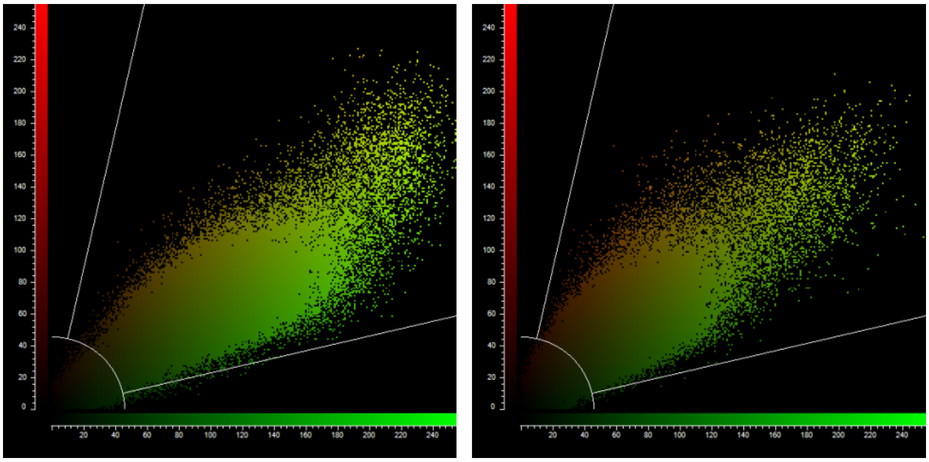

Co-localization of transfected wt BEAF 32B with native BEAF 32

Co-localization of transfected T91A BEAF 32B with native BEAF 32

Flag-tagged wt and T91A BEAF 32B proteins were expressed in S2 cells. The exogenously expressed protein were stained using anti Flag antibody and overlayed with native BEAF 32 stained with anti BEAF antibody. Images were processed using Leica Application Suite Advanced Flourescence software and Pearson’s correlation coefficient was calculated using the software.

Supplementary Figure 3B –

Exogenously expressed mutant BEAF 32B proteins show normal nuclear localization

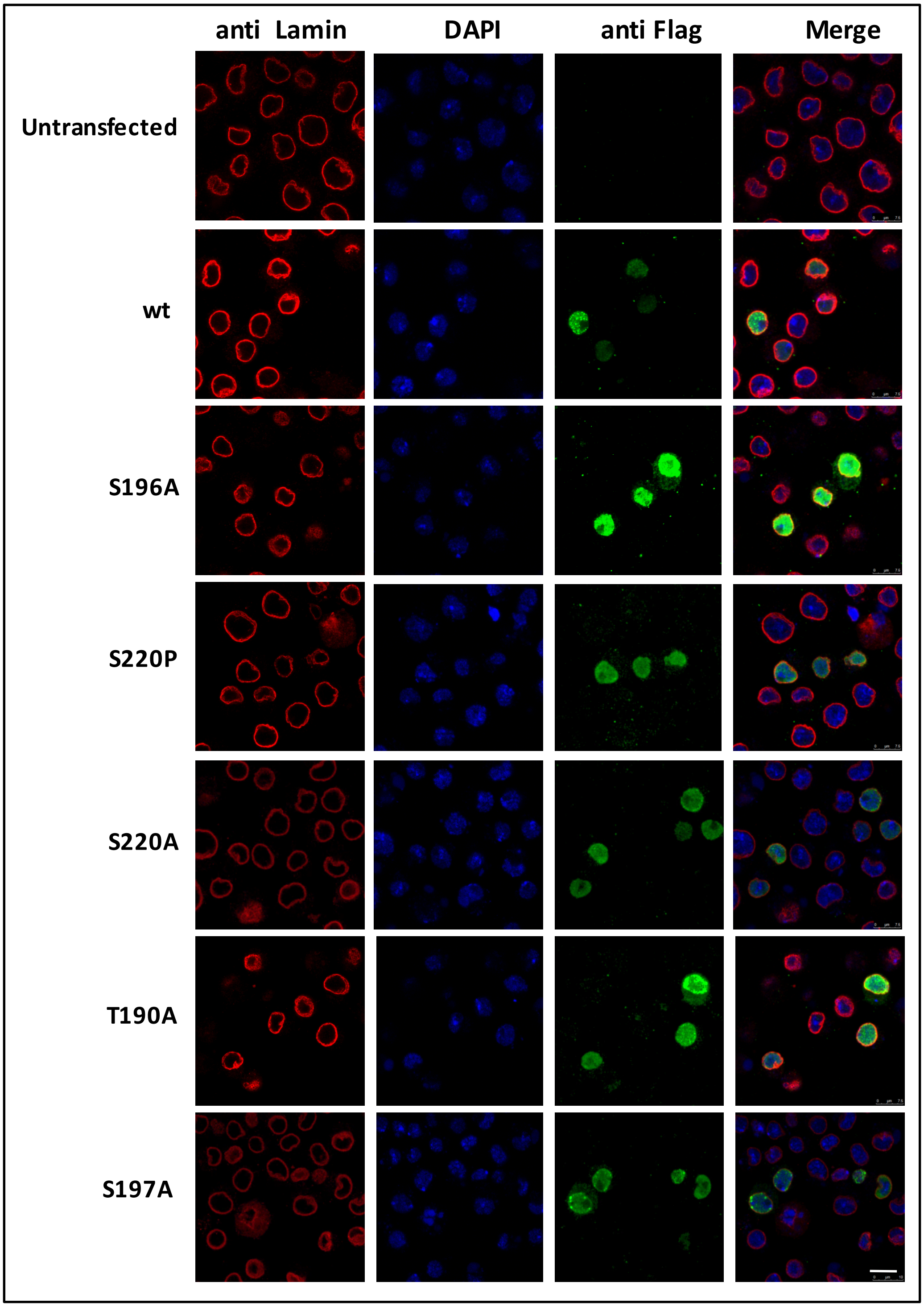

Flag-tagged wt and S196A, S220P, S220A, ST190A, S197A mutant BEAF 32B proteins were expressed in S2 cells, stained with anti Flag antibody and overlayed with nuclei stained with anti Lamin antibody. DNA was stained with DAPI. Scale Bar – 5 µM

Supplementary Figure 4 –

Exogenously expressed mutant BEAF 32B proteins have no effect on boundary activity

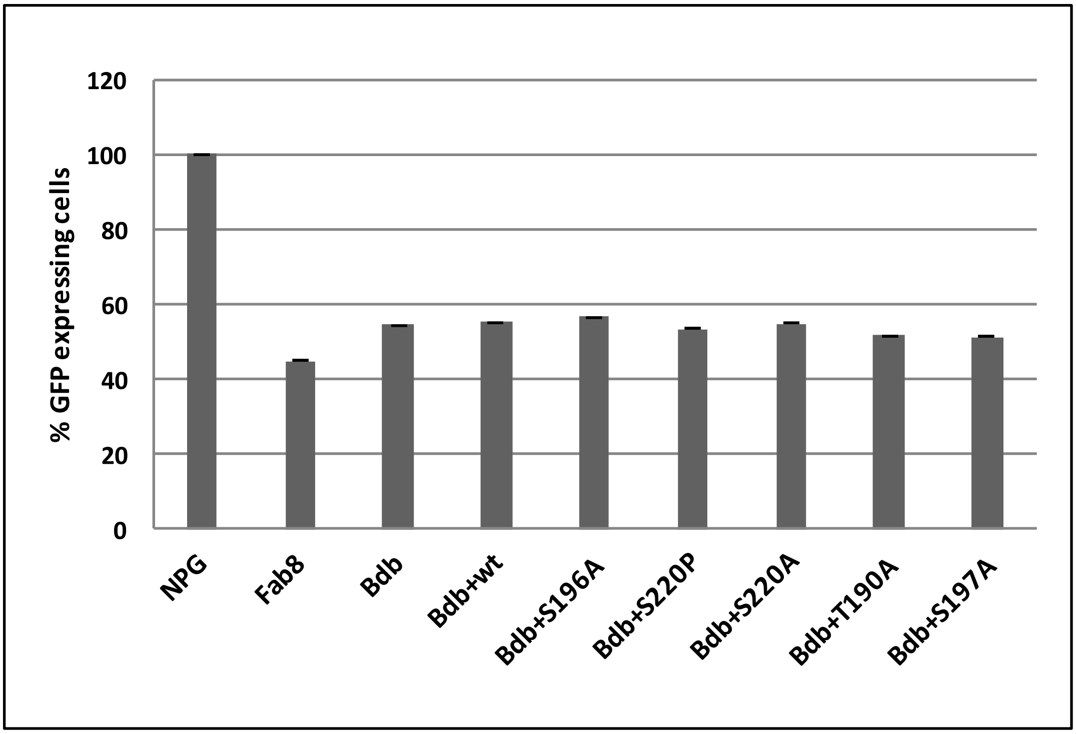

Cell line containing *Bdb* element was transfected with wt, S196A, S220P, S220A ST190A, S197A mutant BEAF 32B proteins. The GFP expressing cells were FACS sorted, counted and plotted as %GFP expressing cells.

Supplementary Figure 5 –

H3K4me3 level decreases if BEAF 32B lacks O-GlcNAcylation

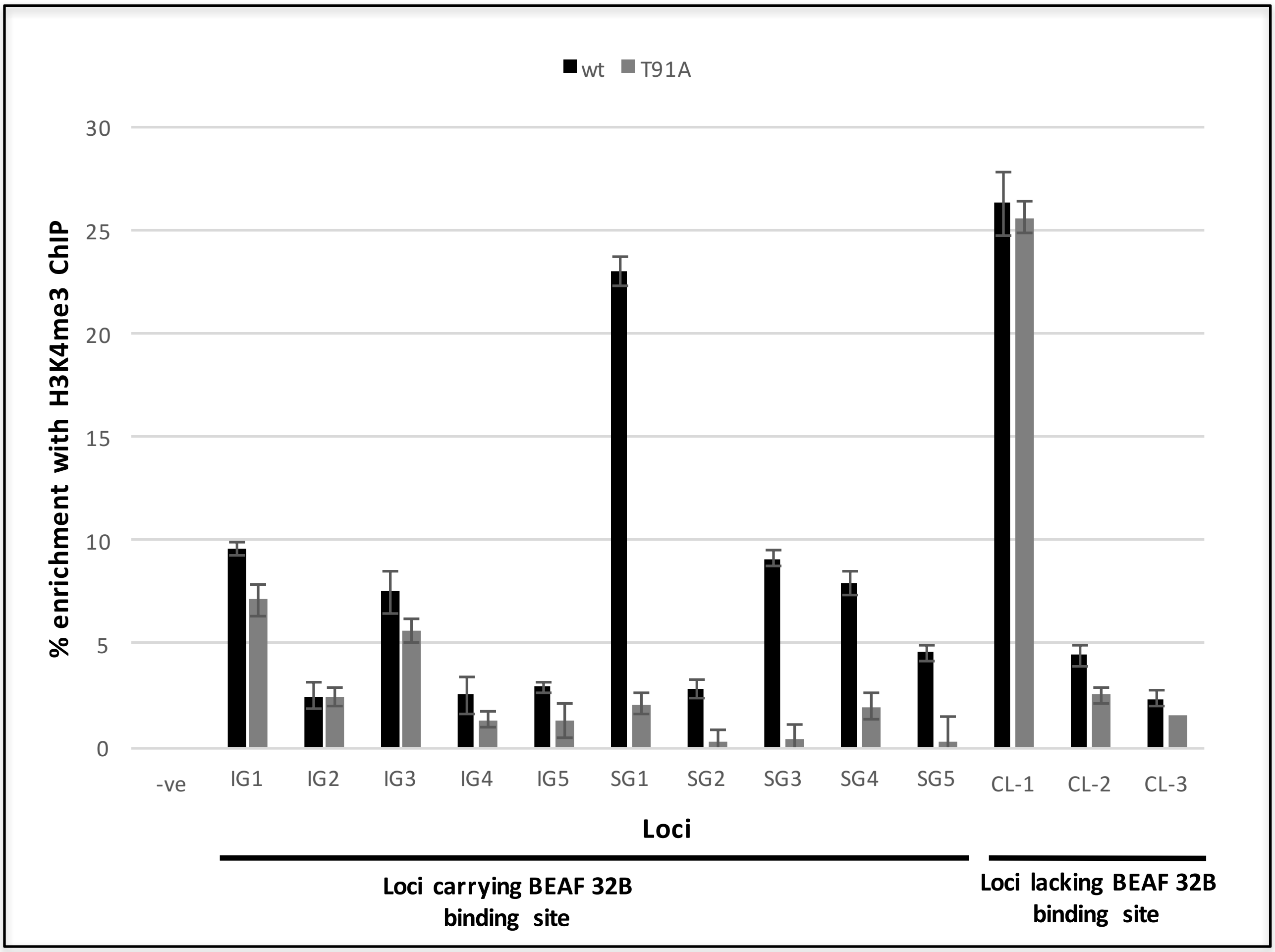

Side by side comparison of H3K4me3 level of loci occupied by BEAF 32B. T91A BEAF 32B which lacks O-GlcNAcylation results in depletion of H3K4me3 mark at the associated loci. The error bar is calculated using the data from three biological replicates.
