# Supplementary material for: O-GlcNAcylation of boundary element associated factor (BEAF 32) in Drosophila melanogaster correlates with active histone marks at the promoters of its target genes
Source: Nucleus. 2017 Sep 14;9(1):65–86. doi: 10.1080/19491034.2017.1367887 (PMC5973196; doi:10.1080/19491034.2017.1367887)
Supplement: Supplementary Files [file kncl-09-01-1367887-s001.zip › Supplementary Tables.docx]

**Supplementary Table 1. Primers used for amplification of BEAF 32 CDS and oligonucleotides used for generating 42bp long dsDNA carrying BEAF 32 binding site (Used in Southwestern blotting’s in Figure 1C and Supplementary Figure 1)**

| BEAF 32 CDS-F | CCCAAGGGTCGTGTTAGC |
| --- | --- |
| BEAF 32 CDS-R | TCTAGAGATGATTGGGATCATCCG |
| BEAF 32 oligo-F | AAAGTCA**CGATA**TTCTTCCACCAAC**CGATA**G**TATCG**CACACT |
| BEAF 32 oligo-R | AGTGTG**CGATA**C**TATCG**GTTGGTGGAAGAA**TATCG**TGACTTT |

**Supplementary Table 2. Primers used for qPCR of 32B occupancy**

| Bdb-F | CGATTGGAGTGCTTAGAGCTG |
| --- | --- |
| Bdb-R | CCCCGACAATCAGATACCC |
| NT-1-F | GGCTTGTTAGGCAGCAATATGAC |
| NT-1-R | TGAACAGTGCCGCTATATGACC |
| NT-2-F | GGAATACCGCACTGTCGTAGG |
| NT-2-R | GCAGCCATCATGGATGTGA |

**Supplementary Table 3. Primers used for qPCR after H3K4me3 ChIP**

| Sl No. | Primer | Sequence |
| --- | --- | --- |
| 1 | -ve_F | TCAGACTTCGCCAATGAGTG |
| 2 | -ve_R | AATGGGTTCGTTAGCACGTC |
| 3 | IG1_L | GAGGTGGTCAGCTGTCGATT |
| 4 | IG1_R | ATTCAGCGGGATCTAAGCAA |
| 5 | IG2_L | GTCCTCGCCATTCACTTTGT |
| 6 | IG2_R | ACTGTCCTCCGGGGTTACTT |
| 7 | IG3_L | AGCGTCAGCATTTCCCATAC |
| 8 | IG3_R | CGGCATTTTGGTTATGCTTT |
| 9 | IG4_L | TTGTGAGACGGCTGTCAAAG |
| 10 | IG4_R | ACTTTGAATCTCGCGGCTTA |
| 11 | IG5_L | TTAAGCTCCTCCCACTGCAT |
| 12 | IG5_R | CGCAATCGAAAAATCAGGTT |
| 13 | SG1_L | TCCGAAAGCTCTTTCAGCAT |
| 14 | SG1_R | AAGAGGAAATCGCCGAGAAT |
| 15 | SG2_L | CGCAGTCTCTGATGGTTTGA |
| 16 | SG2_R | GGCTTCCCCATCAGTGATAA |
| 17 | SG3_L | ACGCGCTAGCTGAAATTGAT |
| 18 | SG3_R | CTTATCATCGCGCACACAGT |
| 19 | SG4_L | CGCAAAGGCAACTCGATTAT |
| 20 | SG4_R | TTCGCCCATCACTAAGGCTA |
| 21 | SG5_L | CAATTGACAAAGCGCAAAGA |
| 22 | SG5_R | TGAACACACCGCCGTTAATA |
| 23 | CL-1_L | GTGGCCGAAACTACGGATTA |
| 24 | CL-1_R | AACCGAGAAATCCGGTAAGG |
| 25 | CL-2_L | CTTGAACTCGTCCGGCTTAC |
| 26 | CL-2_R | TACCGAGGTCCAAGACAAGG |
| 27 | CL-3_L | CGTCTAGGTGAGGCTGATCC |
| 28 | CL-3_R | TGGCTATGGAGGATTTGAGG |

**Supplementary Table 4. Primer used to check cDNA level of genes**

| **Sl No.** | **Primer name** | **Sequence** |
| --- | --- | --- |
| 1 | IG1_CG4710_F1 | ACCATGTGTCCCTCGACTG |
| 2 | IG1_CG4710_R1 | ACGACTTCCGGGTAGCTGAT |
| 3 | IG2_ CG9326_F3 | CCAGGAACTGATGCCTTTTG |
| 4 | IG2_ CG9326_R3 | CATACAACGACGCTAGGGACT |
| 5 | IG3_CG5094_F3 | CGGACAACGAGGTGTACAAG |
| 6 | IG3_CG5094_R3 | TTCTGAAGCATCGACAGCAG |
| 7 | IG4_ CG1582N_F2 | GCAAGCTGATGCTGTATGGA |
| 8 | IG4_ CG1582N_R2 | ACGGTCAAATGATCGCTGTT |
| 9 | IG5_CG9577N_F1 | CCTCTTCCCAGACAAGGATT |
| 10 | IG5_CG9577N_R1 | TTGAGCTTGTTCAGCAGGAG |
| 11 | IG6_CG4144N_F2 | TACGGGGACGACTTTCACAA |
| 12 | IG6_CG4144N_R2 | ATCGTCGAAGTTCAGGAAGC |
| 17 | SG1_ CG14073_F2 | CTCTGTCCGAACAACAGCAA |
| 18 | SG1_ CG14073_R2 | TTTGATGAGCACAACCTTCG |
| 19 | SG2_CG12717N_F2 | AGGGACGTTGATGAAGATGC |
| 20 | SG2_CG12717N_R2 | TCCTTGATGGTGATGCTCAG |
| 21 | SG3_CG1837N_F2 | AAGACACCTTTGCCAAGCAC |
| 22 | SG3_CG1837N_R2 | AACTGGGTGCAGTCGATCTT |
| 23 | SG4_ CG15811_F2 | TGCCCAGGCTTATTGTCTTC |
| 24 | SG4_ CG15811_R2 | TTCGAGAGACTGCCCAAATC |
| 25 | SG5_CG42803N_F2 | CGGATATGTTATGGCCGATAGT |
| 26 | SG5_CG42803N_R2 | TTCGCCTGGTCTTATCCAAT |
| 27 | SG6_CG7425N_F1 | CCAAACATCAACAGCAATGG |
| 28 | SG6_CG7425N_R1 | TCTCGTGCCAGCTCATTGTA |
| 29 | SG7_CG9045N_F2 | AGATATGGATCTGCGGGCTA |
| 30 | SG7_CG9045N_R2 | GCAGCAAGTGCCTTTTTGA |
| 31 | SG8_CG9198N_F2 | ACCAGGTGATGCTGCTATTCT |
| 32 | SG8_CG9198N_R2 | TCCTGGCTGCGACTCAAATA |
| 33 | SG9_CG9774N_F2 | TTCCACGCATATTCCAGCTA |
| 34 | SG9_CG9774N_R2 | TGTGAACGAACTCGTGACCT |
| 35 | SG10_CG7833N_F2 | AGCTGCCCTACTATGCCAAA |
| 36 | SG10_CG7833N_R2 | GTCCCAAAGTGGTGGACATT |
| 37 | ACT5CF | GTGGATCTCCAAGCAGGAGTAC |
| 38 | ACT5CR | CTCGCACTTGCACTTTCGCTGC |
